# GPR30 Selective Agonist G1 Exhibits Antiobesity Effects and Promotes Insulin Resistance and Gluconeogenesis in Postmenopausal Mice Fed a High-Fat Diet

**DOI:** 10.1155/2024/5513473

**Published:** 2024-11-08

**Authors:** Da Liu, Mingqi Zheng, Congcong Lu, Mengdan Miao, Yinge Zhan, Fangfang Ma, Yajuan Yin, Mei Wei, Wei Wang, Wenyao Wang, Xiangbin Meng, Jing Li, Yaohua Zhang, Gang Liu, Yi-Da Tang

**Affiliations:** ^1^Department of Cardiology, The First Hospital of Hebei Medical University, Hebei Key Laboratory of Cardiac Injury Repair Mechanism Study; Hebei Key Laboratory of Heart and Metabolism; Hebei Engineering Research Center of Intelligent Medical Clinical Application; Hebei International Joint Research Center for Structural Heart Disease, Shijiazhuang, China; ^2^Graduate School of Hebei Medical University, Shijiazhuang, China; ^3^Department of Cardiology and Institute of Vascular Medicine, Peking University Third Hospital; Key Laboratory of Molecular Cardiovascular Science, Ministry of Education, Beijing, China

**Keywords:** fat mobilization, gluconeogenesis, hormone replacement therapy, insulin resistance, pyruvate metabolism

## Abstract

**Background:** G1, a specific agonist targeting the G protein–coupled receptor 30 (GPR30), has demonstrated significant involvement in combating obesity and regulating glucose homeostasis. Nevertheless, the beneficial effects of G1 treatment have solely been investigated in animal models under normal feeding conditions, leaving its therapeutic potential in high-fat feeding scenarios unexplored.

**Material and Methods:** To address this gap, our study employed an ovariectomized high-fat diet mouse model to assess the therapeutic effects of G1 in combating obesity and metabolic dysfunction.

**Results:** The findings revealed that G1 treatment resulted in weight loss, but concurrently led to increased blood glucose levels and insulin resistance. Treatment with G1 resulted in an amplification of fat mobilization and an enhancement of pyruvate carboxylase activity in mice fed a high-fat diet. Moreover, the combined impact of G1 treatment and a high-fat diet on pyruvate metabolism, as well as the regulation of crucial gluconeogenesis enzymes such as pyruvate dehydrogenase kinase 4 (PDK4), phosphoenolpyruvate carboxykinase (PEPCK), and glucose transporter 2 (GLUT2), expedites the elevation of blood glucose and the progression of insulin resistance.

**Conclusions:** These findings indicate that G1 treatment is influenced by a high-fat diet, potentially disrupting glucolipid metabolism and promoting insulin resistance alongside its antiobesity effects. Consequently, further investigation is imperative to thoroughly explore this potential toxic side effect of G1 therapy.

## 1. Introduction

Obesity is a global health concern closely associated with an increased risk of various chronic diseases, such as cardiovascular disorders, diabetes, and cancers [1]. As such, combating or reversing obesity has become a global challenge [2]. GPR30 (GPER), recognized as an estrogen membrane receptor, has its agonists confirmed to inhibit weight gain in obese mice and ameliorate metabolism [3, 4]. When activated in the pancreas, adipose tissue, liver, and skeletal muscles, GPR30 functions by inhibiting lipid synthesis and promoting lipid oxidation, reducing lipid accumulation [5, 6]. Moreover, GPR30 plays a role in blood glucose regulation by enhancing insulin secretion, improving glucose uptake, and reducing glucose production in nonadipose tissues such as the pancreas, skeletal muscle, and liver [7]. Additionally, the absence of GPR30 results in obesity, increased insulin resistance, and metabolic dysfunction in mice [8]. Therefore, activating GPR30 emerges as a promising new target for preventing weight gain and lipid accumulation.

The discovery of GPR30 agonists and antagonists has significantly deepened our understanding of GPR30's function in physiology and disease. Activating GPR30 has been shown to inhibit weight gain and improve metabolism. Administration of G1 (G-1), a GPR30 receptor agonist with the chemical formula C_21_H_18_BrNO_3_, to ovariectomized (OVX) estrogen-deficient mice results in weight reduction, decreased fat content, enhanced energy expenditure, and increased utilization of brown adipose tissue [9]. Preclinical studies suggest that G1 may prevent obesity through various mechanisms, such as promoting lipolysis and oxidation, inhibiting adipocyte proliferation and differentiation, and regulating the insulin signaling pathway [3]. Furthermore, G1 activation of GPR30 has improved glucose tolerance in OVX mice, maintaining glucose homeostasis by lowering fasting blood glucose and insulin levels [5]. Thus, G1 shows potential as a novel therapeutic for obesity and diabetes.

Research indicates that postmenopausal women experience decreased energy expenditure, increased fat deposition, insulin resistance, and impaired glucose and lipid metabolism [10, 11]. This leads to a higher incidence of obesity and insulin resistance (metabolic syndrome) in women compared to age-matched men [12]. One reason could be that premenopausal women's estrogen levels offer protective effects against metabolic disorders. However, postmenopausal women lose the protective effects of estradiol (E2), leading to reduced insulin sensitivity in pancreatic *β*-cells, resulting in abnormal lipid metabolism, pathological obesity, and manifestations of metabolic syndrome. Studies have found that treating postmenopausal obese women with G1 activating the GPR30 receptor yields significant results [13–15].

However, dietary habits also play a vital role in the onset and progression of obesity. An imbalance between energy intake and expenditure is a primary reason for fat storage and weight gain [16]. High-fat diet (HFD)-induced obesity in mice more aptly simulates the obesity observed in modern patients consuming excessive fat and sugar [16, 17]. Given the noticeable weight loss effects post GPR30 receptor activation, we introduced a HFD as an intervention. In this study, we report the therapeutic effects of the GPR30 agonist G1 on menopausal mice on a HFD. Moreover, in our experiments, we unexpectedly found that administering G1 treatment under a HFD led to reduced glucose tolerance and increased insulin resistance in OVX mice, despite weight reduction. No studies have reported similar findings. Hence, we believe that the weight-reducing effects of GPR30 receptor activation might manifest side effects under HFD high-fat dietary intervention.

## 2. Materials and Methods

### 2.1. Animal Models

Female 8-week-old C57BL/6 mice, weighing between 2 and 24 g, were procured from Skbex Biotechnology (China). These mice were randomly allocated into four experimental groups, each comprising 10 animals (*n* = 10). Further details on the grouping and experimental protocols can be found in the Supporting Information section.

### 2.2. Group Design

The experimental setup was divided into four distinct groups: the sole ovariectomy group, the ovariectomy followed by the G1 treatment group, the ovariectomy followed by a HFD group, and the ovariectomy followed by a HFD plus G1 treatment group. All groups underwent ovariectomy to negate the effects of estrogen. The study is aimed at demonstrating the therapeutic efficacy of G1 by comparing the simple OVX group against the G1-treated group and assess the intervention of a HFD on OVX mice by comparing them against the HFD groups. Additionally, the comparative efficacy of G1 treatment under varying dietary conditions is investigated by contrasting the G1 treatment groups.

### 2.3. Oral Glucose Tolerance Test (OGTT)

Following an overnight fast of at least 12 h, both normal and diabetic rats were administered 20% glucose solution intragastrically. Orbital blood samples were collected at intervals of 0, 30, 60, 90, and 120 min postadministration. Blood glucose levels were measured using a BS-180 automatic biochemical analyzer. Refer to the Supporting Information section for a detailed methodology.

### 2.4. Sample Collection and Processing

Refer the Supporting Information section for detailed protocols on sample collection and processing.

### 2.5. RNA Isolation, cDNA Synthesis, and Real-Time Quantitative PCR

For detailed methodology on RNA isolation, cDNA synthesis, and real-time quantitative PCR, refer to the Supporting Information section.

### 2.6. Western Blot Analysis

The protocols for protein extraction, electrophoresis, and immunoblotting for Western Blot analysis are detailed in the Supporting Information section.

### 2.7. Screening for Obesity-Associated Genes in Menopausal Women

The study utilized metabolic pathway gene (MRG) sets for *Homo sapiens* from the Reactome website. Obese female data set GSE151839 was downloaded from the GEO database, excluding skin tissue samples and retaining only adipose tissue data. Boxplots and PCA plots were employed to standardize expression data and to discern differences between the obese and normal groups. A stringent criterion (*p* ≤ 0.05 and logFC ≥ ±0.5) was adopted to select DEGs, which were then identified as obesity-related genes in females. The intersection of MRGs and DEGs was defined as obesity-associated metabolic genes (OMGs) and further analyzed for Gene Ontology (GO) and Kyoto Encyclopedia of Genes and Genomes (KEGG) enrichment.

### 2.8. Screening of HUB Genes

DEGs and MRGs were selected through the jvenn map Wayne tool, and intersection results were imported into the STRING database for analysis. The Top 10 HUB genes were identified using Cytoscape software.

### 2.9. Screening of Core Genes

A weighted gene coexpression network was constructed using the “WGCNA” package in R software to explore the interaction among differentially expressed genes (DEGs) in obese elderly female samples. Data standardization, sample exclusion, and Pearson's correlation coefficient calculations were performed according to the procedure outlined in the Supporting Information section.

### 2.10. Elisa

For detailed methodology on enzyme-linked immunosorbent assay, refer to the Supporting Information section.

### 2.11. Morphometric Analyses

Refer to the Supporting Information section for detailed protocols on morphometric analyses.

### 2.12. Liver Tissue Mitochondrial Extraction

The procedure for the extraction of mitochondria from liver tissues is described in the Supporting Information section.

### 2.13. Homeostasis Model Assessment of Insulin Resistance (HOMA-IR)

Refer to the Supporting Information section for a detailed methodology on HOMA-IR.

### 2.14. Statistical Analysis

Data were analyzed using SPSS 18.0 software (IBM Corporation, United States). Comparisons among three or more groups were conducted using One-way ANOVA, and comparisons between two groups were conducted using the unpaired *t*-test. A *p* value < 0.05 was considered statistically significant. All data are presented as mean ± standard deviation (*χ* ± *S*). GraphPad Prism 7.0 software was utilized for generating figures.

## 3. Results

### 3.1. G1 Treatment Accelerates Insulin Resistance in OVX Mice on a HFD

**Figure 1:**
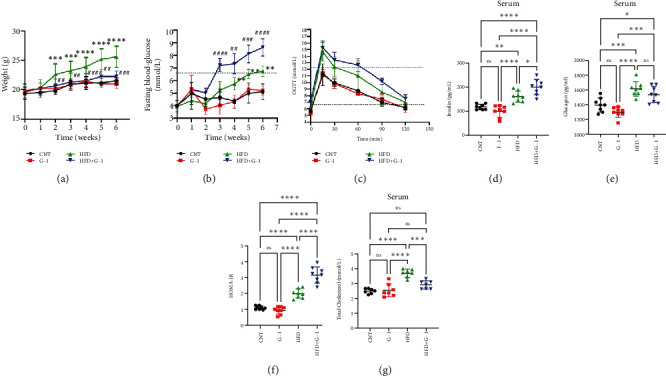
G1 reduced body weight and promoted insulin resistance in high-fat OVX mice. Four groups were divided into a high-fat diet and a control diet, which were interleaved with G1 treatment and placebo. (a) Changes in body weight over time in each group (*n* = 6). (b) Changes of fasting blood glucose in each group over time (*n* = 6). (c) Oral glucose tolerance test (OGTT) in each group (*n* = 6). (d) Fasting insulin level in four groups (*n* > 6). (e) fasting glucagon level in four groups (*n* > 6). (f) Insulin resistance index in four groups (*n* > 6). (g) liver triglyceride levels in four groups (*n* > 6). (a, b) Two-way ANOVA; ⁣^∗^*p* < 0.05, ⁣^∗∗^*p* < 0.01, ⁣^∗∗∗^*p* < 0.001, ⁣^∗∗∗∗^*p* < 0.0001 for HFD group vs. CON group; #*p* < 0.05, ##*p* < 0.01, ###*p* < 0.001, ####*p* < 0.0001 for HFD+G1 group versus HFD group. (d–g) One-way ANOVA with Bonferroni posthoc test; ns > 0.05, ⁣^∗^*p* < 0.05, ⁣^∗∗^*p* < 0.01, ⁣^∗∗∗^*p* < 0.001, and ⁣^∗∗∗∗^*p* < 0.0001.

In this study, we investigated the effects of G1 treatment on glucose and lipid metabolism in OVX mice maintained on a HFD. Building on previous findings that G1 positively influences adipose tissue mobilization and weight management in obese OVX mice on a standard diet, we divided OVX mice into four groups: control chow, control chow + G1, HFD, and HFD + G1. Our observations revealed that the body weight of mice receiving G1 treatment alongside an HFD did not increase significantly over time, contrasting with the significant weight gain observed in the HFD group by the 6th week (*p* < 0.01, Figure 1(a)). These findings suggest that G1 possesses intrinsic antiobesity properties.

However, a notable trend emerged when examining fasting blood glucose levels. The HFD + G1 group exhibited a significant increase in fasting blood glucose starting from the second week, differing markedly from the gradual rise observed in the HFD group (Figure 1(b)). OGTTs further indicated the potential onset of glucose intolerance or insulin resistance (Figure S5C, D) mellitus in the HFD + G1 group, evidenced by elevated initial blood glucose levels and delayed glucose clearance (Figure 1(c)). Correspondingly, insulin levels were significantly higher in this group (Figure 1(d)). Calculations of the insulin resistance index confirmed increased insulin resistance in mice fed an HFD, with the effect being particularly pronounced in the G1-treated group (Figure 1(f)). Additionally, glucagon levels were elevated across all HFD groups, although they were slightly decreased in the G1-treated group (Figure 1(e)).

### 3.2. G1 Therapy Promotes Lipolysis and Fat Utilization

**Figure 2:**
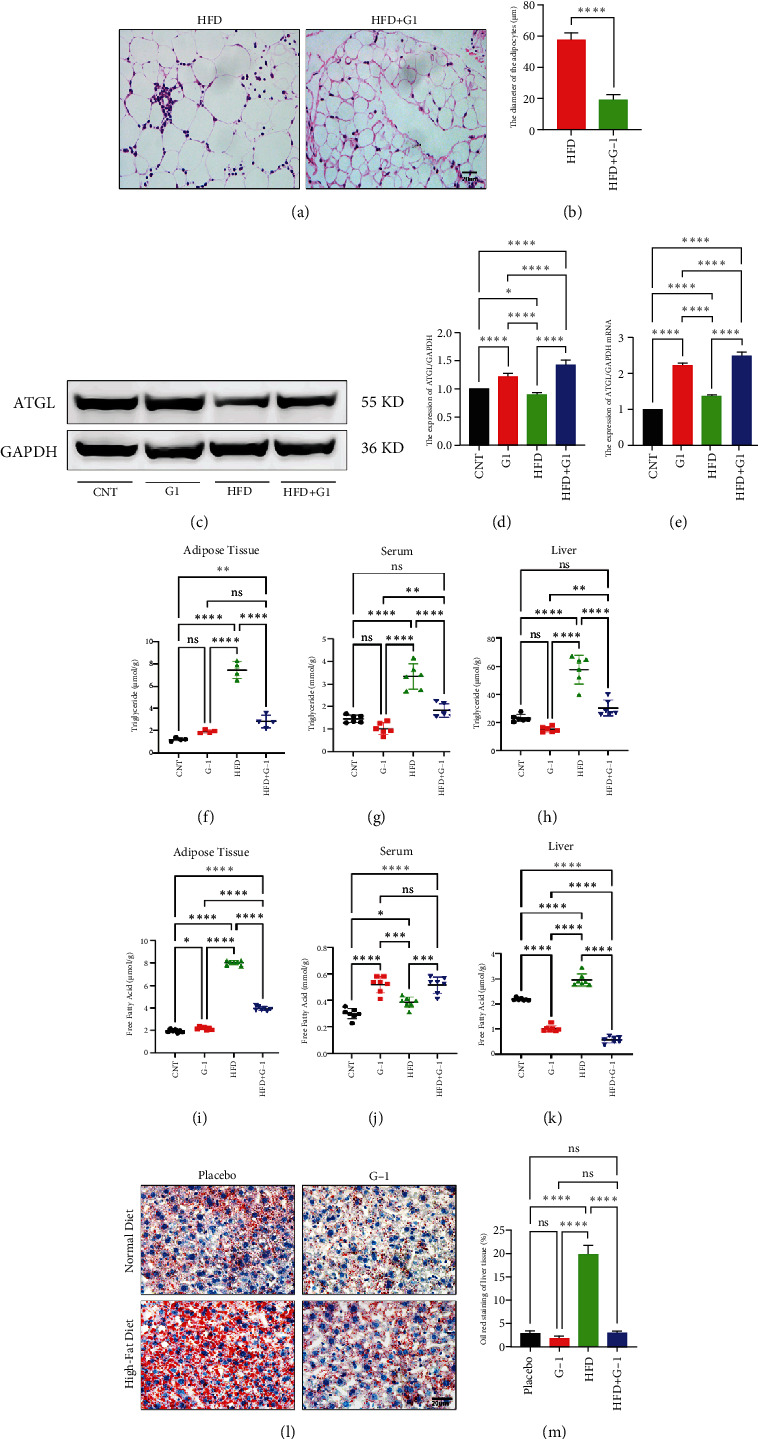
G1 treatment promoted lipolysis and utilization. (a, b) Comparison of visceral adipocyte diameter size in the high-fat and high-fat plus G1 groups. (c–e) ATGL transcription and translation in adipose tissues. (f–h) Adipose tissue and triglyceride levels in serum and liver (*n* = 6). (i–k) Adipose tissue and fatty acid levels in serum and liver (*n* = 6). (l and m) Oil red O staining of liver tissue to observe lipid accumulation. One-way ANOVA with Bonferroni post hoc test; ⁣^∗^*p* < 0.05, ⁣^∗∗^*p* < 0.01, ⁣^∗∗∗^*p* < 0.001, and ⁣^∗∗∗∗^*p* < 0.0001.

We evaluated the impact of G1 therapy on fat mobilization by measuring triglycerides (TGs) and free fatty acids (FFA) in adipose tissue, serum, and liver. G1 treatment significantly reduced TG content in adipose tissue and promoted lipolysis, as evidenced by increased FFA levels in G1-treated mice on an HFD (Figures 2(f) and 2(i)). Serum analyses supported these findings, showing decreased TG and increased FFA levels in G1-treated mice (Figures 2(g) and 2(j)). Liver tissue analysis revealed reduced TG content and lipid accumulation in mice treated with G1 and fed an HFD (Figures 2(h) and 2(k)). Additionally, adipocyte diameter was significantly smaller in G1-treated mice on an HFD (Figures 2(a) and 2(b)), reinforcing the notion of enhanced lipid mobilization. Western blot analysis of adipose triglyceride lipase (ATGL), hormone-sensitive lipase (HSL), and perilipin further supported increased lipolysis in G1-treated groups (Figures 2(c), 2(d), and 2(e), Figure S5A–C). Furthermore, Oil Red O staining of liver sections demonstrated a marked reduction in lipid accumulation in G1-treated groups (Figures 2(l) and 2(m)).

### 3.3. Identification of Metabolically Relevant Key Genes in Obese Postmenopausal Women

**Figure 3:**
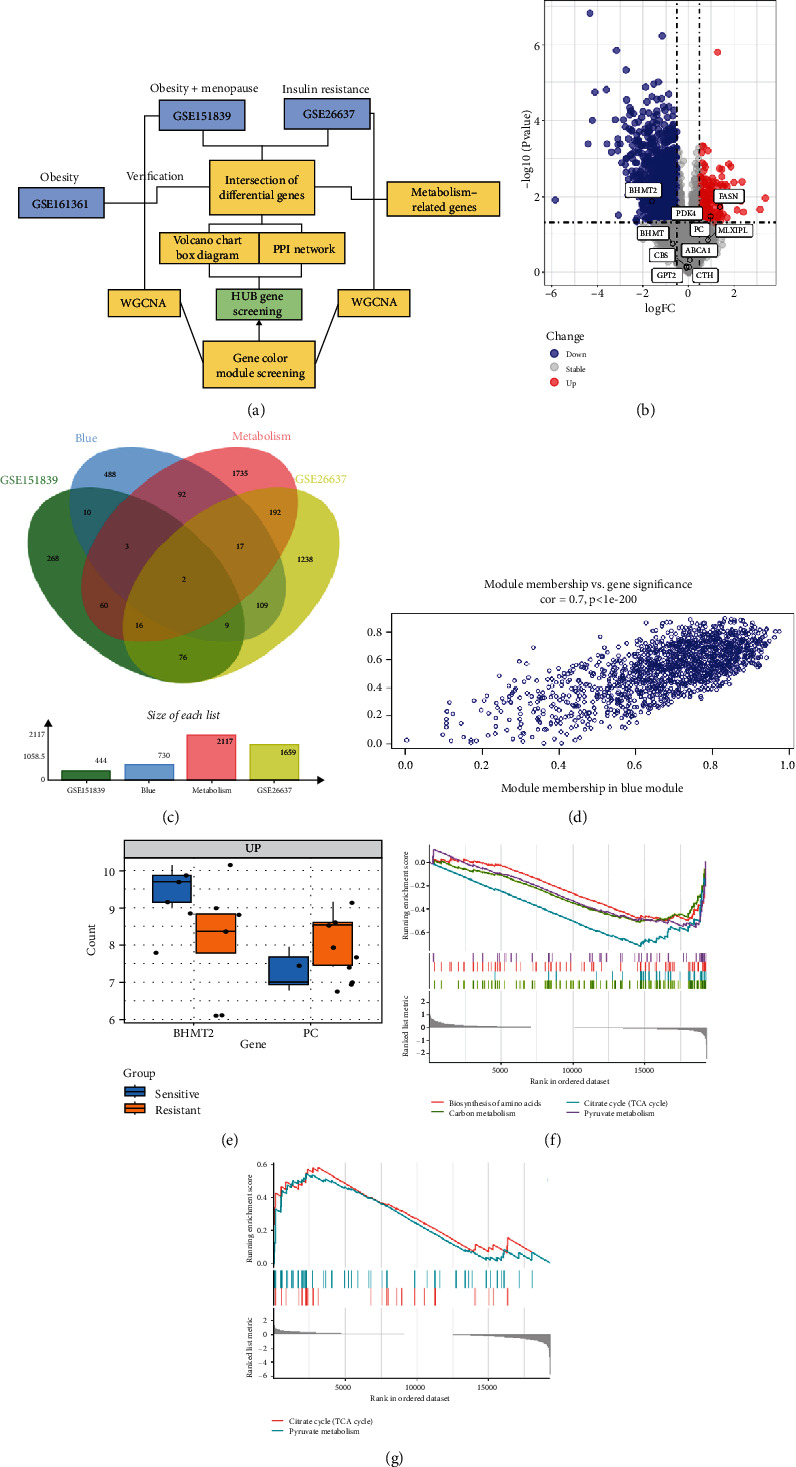
Bioinformatics was used to predict differentially expressed genes in obese or insulin-resistant patients. (a) bioinformatics analysis flow chart. (b) The core genes were shown on the volcano map in the GSE26637 dataset. (c) The intersection genes result from the data of GSE151839, BLUE module, metabolism, and GES26637. (d) Scatter plot describing the relationship between MM and GS in the blue module. (e) Boxplot of core genes in the GSE26637 dataset. (f) Determination of PEP content in the liver. (g) The amount of pyruvate in the liver.

To understand the modifications in HFD-induced insulin resistance potentially mediated by G1 treatment, we analyzed adipose tissue samples from obese and normal-weight postmenopausal women. Differential gene expression analysis identified 444 DEGs, of which 81 were directly related to metabolism (Figure S1B–D). Protein–protein interaction network analysis revealed 10 hub genes; intriguingly, 8 of these were downregulated in obese postmenopausal women, with the exceptions of BHMT and BHMT2 (Figure S1E, F). KEGG pathway analysis suggested the downregulation of glycolipid metabolism pathways (Figure S1H). Moreover, weighted gene coexpression network analysis (WGCNA) identified BHMT2 and PC (pyruvate carboxylase) as the most metabolically relevant hub genes in this demographic (Figures 3(a), 3(d), and 3(e)).

### 3.4. Validation of Key Genes Using the GSE26637 Dataset

**Figure 4:**
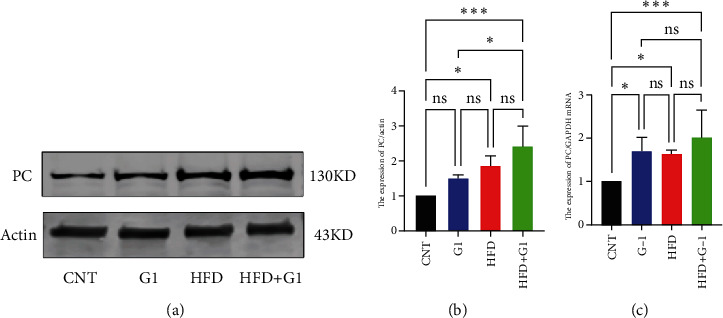
Verification of PC gene. (a, b) Protein verification of pyruvate carboxylase. (c) PCR validation of pyruvate carboxylase. ⁣^∗^*p* < 0.05, ⁣^∗∗^*p* < 0.01, ⁣^∗∗∗^*p* < 0.001, and ⁣^∗∗∗∗^*p* < 0.0001.

To validate our findings, we cross-referenced them with the GSE26637 dataset. Comparative analysis revealed that PC and BHMT2 were consistently associated with obesity and insulin resistance (Figures 3(b), 3(c), and 3(e)). The altered expression levels of PC and BHMT2 suggest a potential link to the development of insulin resistance in obese individuals. Gene set enrichment analysis (GSEA) further confirmed a significant correlation between the PC gene and metabolic pathways in insulin-resistant obese women (Figure 3(g)). In our insulin resistance model induced by an HFD, we observed a notable increase in PC expression. G1 treatment led to a modest upregulation of PC expression, and importantly, G1 treatment markedly enhanced PC expression levels in mice on an HFD. These findings were confirmed through Western blotting and quantitative PCR analyses (Figures 4(a), 4(b), and 4(c)).

### 3.5. Effect of G1 Treatment on Key Proteins of the Pyruvate Metabolic Pathway

**Figure 5:**
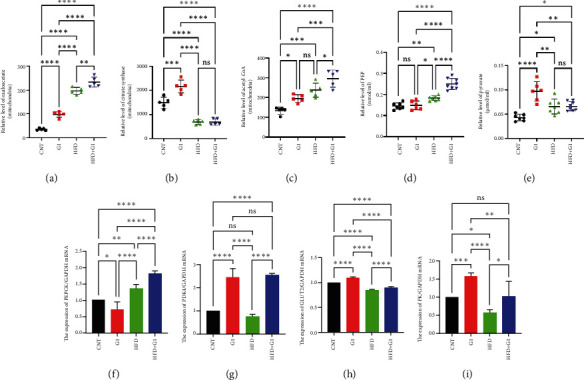
G1 promotes gluconeogenesis in high-fat diet mice. (a) OAA content in liver mitochondria; (b) contents of citrate synthase (CS) in liver mitochondria; (c) the content of acetyl-CoA in liver mitochondria; (d) determination of PEP content in the liver; (e) the amount of pyruvate in the liver; (f) transcription levels of PEPCK mRNA in the liver; (g) transcription level of PDK4 mRNA in the liver; (h) Transcription level of GLUT2 mRNA in the liver; (i) PK mRNA transcription level in the liver; ⁣^∗^*p* < 0.05, ⁣^∗∗^*p* < 0.01, ⁣^∗∗∗^*p* < 0.001, and ⁣^∗∗∗∗^*p* < 0.0001.

We examined the impact of G1 treatment on key proteins within the pyruvate metabolic pathway. Levels of acetyl-CoA and oxaloacetate (OAA), crucial components of the tricarboxylic acid (TCA) cycle, were elevated in G1-treated mice, suggesting enhanced metabolic activity within these pathways. Additionally, the expression of citrate synthase (CS), an important enzyme in the TCA cycle, was downregulated under HFD conditions. This downregulation was attenuated in the presence of G1 treatment, and an upregulation was observed in the group treated with G1 alone (Figures 5(a) and 5(b)).

### 3.6. Effects of G1 Treatment on Key Enzymes and Metabolites in the Hepatic Gluconeogenesis Pathway

We investigated the impact of G1 treatment on markers of the gluconeogenesis pathway in mice fed an HFD. Notably, levels of phosphoenolpyruvate carboxykinase (PEPCK) and phosphoenolpyruvate (PEP) were elevated, indicating increased gluconeogenic activity, especially pronounced in the HFD + G1 group (Figures 5(d) and 5(f)). Additionally, pyruvate levels were significantly higher in both the HFD and G1-treated groups (Figure 5(e)).

### 3.7. G1 Treatment Upregulates Pyruvate Dehydrogenase Kinase 4 (PDK4) Expression, Inhibiting TCA Cycle Activity

Our analysis revealed that G1 treatment leads to upregulation of PDK4 mRNA levels. This upregulation suggests reduced activity of the TCA cycle, consequently promoting gluconeogenesis. In the HFD + G1 group, there was a notable increase in PDK4 expression compared to the HFD group (Figure 5(g)).

### 3.8. G1 Enhances Glucose Transport and Promotes Hepatic Glucose Homeostasis in HFD Mice

Finally, we assessed the expression levels of glucose transporter 2 (GLUT2) and pyruvate kinase (PK) transcripts under G1 treatment. We observed significant upregulation of these transcripts in G1-treated mice, particularly in the HFD + G1 group, suggesting enhanced glucose transport and improved hepatic glucose homeostasis under HFD conditions (Figures 5(h) and 5(i)).

## 4. Discussion

This study presents the novel finding that the therapeutic effects of G-1 vary significantly between HFD and a normal diet (ND) in OVX mice, indicating a complex interplay among diet, metabolic regulation, and pharmacological intervention. Our results demonstrated that G-1 significantly reduced body weight in HFD mice, which is consistent with its known role as a specific agonist of the G protein–coupled estrogen receptor (GPER) [5, 18]. These findings align with previous studies indicating the involvement of GPER in regulating body weight and adiposity. However, the impact of G-1 on ND mice was minimal, suggesting that the presence of an obesity-inducing diet is a crucial factor in G-1's therapeutic efficacy.

Interestingly, although G-1 exhibited antiobesity effects, it simultaneously increased fasting blood glucose levels, impaired glucose tolerance, and elevated the insulin resistance index in HFD mice. These adverse effects were accompanied by increased mobilization of fatty acids, suggesting enhanced lipid metabolism under HFD conditions. The underlying mechanism appears to involve accelerated *β*-oxidation of fatty acids and increased gluconeogenesis, possibly due to G-1's modulation of metabolic pathways in the liver [19, 20]. These observations highlight a potential deleterious effect of G-1 under HFD conditions, which could undermine its therapeutic benefits.

Conversely, other studies have reported beneficial effects of G-1 on glucose homeostasis, suggesting that specific experimental conditions—including diet type and timing of intervention—significantly influence G-1's metabolic outcomes [18]. For instance, Sharma et al. found that G-1 treatment in OVX mice reduced fasting blood glucose and insulin levels and improved glucose tolerance, thereby reducing HOMA-IR and enhancing peripheral insulin action to maintain glucose homeostasis. However, in their study, G-1 treatment started after inducing obesity with an HFD, and the mice were fed an ND during the treatment period [21]. In contrast, our study administered G-1 immediately after ovariectomy and continued HFD feeding for 6 weeks during treatment. We observed that G-1 treatment did not significantly affect blood glucose levels in ND-fed mice, unlike in HFD-fed mice. This suggests that dietary context and timing of G-1 administration are critical factors influencing its metabolic effects.

The molecular mechanisms underpinning the observed metabolic effects of G-1 are multifaceted. G-1's activation of GPER is known to influence various signaling pathways, including those involved in insulin secretion and lipid metabolism [22]. Under normal dietary conditions, G-1 can activate the ERK and PI3K pathways to regulate blood glucose levels, maintaining glucose and lipid homeostasis [6]. Interestingly, the activation of the ERK and PI3K pathways is mutually exclusive, which may help balance glucose levels and insulin secretion. However, under an HFD, this balance appears disrupted, leading to increased fatty acid mobilization and altered glucose metabolism. Specifically, our study suggests that G-1 enhances fatty acid *β*-oxidation and activates PC, driving the gluconeogenic pathway and increasing blood glucose levels.

Targeting the interaction between ATGL, HSL, and perilipin, ATGL is the primary enzyme that initiates lipolysis by hydrolyzing triglycerides into diglycerides and free fatty acids, playing a crucial role in both short-term and long-term energy regulation [22]. Following ATGL's action, HSL further catalyzes the breakdown of diglycerides into monoglycerides and free fatty acids and also has some capacity to hydrolyze monoglycerides, releasing additional free fatty acids. Perilipin is a regulatory protein on the surface of lipid droplets that helps protect them from lipase attack [23]. When stored fat needs to be mobilized, the phosphorylation state of perilipin changes, allowing ATGL and HSL to access and break down the lipids within the droplets. Regarding hormonal regulation, epinephrine regulates HSL activity and ATGL by phosphorylating perilipin and activating protein kinase A (PKA), thereby promoting the release of fatty acids [24]. In our study, we found that ATGL protein expression was upregulated in the G1-treated group, suggesting that G1 promotes the breakdown of triglycerides, especially in the context of a HFD. This upregulation of ATGL anp may be the main reason for the accumulation of lipid droplets in adipose tissue. The results showed that G1 treatment increased the phosphorylation level of perilipin, while HSL expression did not show significant changes.

Through literature review, we learned that when GPR30 is activated, it may regulate various intracellular functions through multiple pathways such as the cAMP signaling pathway, PI3K/Akt signaling pathway, or MAPK signaling pathway [25]. Literature indicates that GPR30 activation can increase cAMP levels, which is a key molecule for activating PKA, and PKA can phosphorylate perilipin [26]. Therefore, the activation of GPR30 promotes the phosphorylation of perilipin through this mechanism, thereby affecting fatty acid mobilization. Studies have also shown that the phosphorylation of perilipin is closely related to fatty acid release; it facilitates enzymes such as ATGL and HSL to access fatty acids for lipolysis by regulating the surface structure of lipid droplets [27].

Moreover, our research delved into the changes in the expression and activity of key metabolic enzymes, including ATGL and PEPCK. Despite the reduction in circulating free fatty acids and hepatic lipid deposition, G1 treatment led to increased acetyl-CoA levels and PC activity, contributing to elevated blood glucose levels and insulin resistance in HFD mice. These findings suggest that while G1 promotes lipolysis through upregulating ATGL and promoting perilipin phosphorylation, it also influences gluconeogenic pathways via increased PEPCK expression and PC activity.

These results reveal a paradoxical effect of G1, wherein it both ameliorates lipid accumulation and aggravates glucose metabolism disorders depending on the dietary environment. The increased acetyl-CoA levels may enhance gluconeogenesis, leading to elevated blood glucose and insulin resistance. Therefore, our findings highlight the complex metabolic effects of G1 treatment, indicating that it accelerates lipolysis by upregulating ATGL and promoting perilipin phosphorylation but also affects glucose metabolism in a way that may require careful consideration in therapeutic contexts.

Research has shown that a HFD can negatively impact the expression and activity of the PI3K/Akt signaling pathway [28]. Chronic consumption of an HFD is associated with insulin resistance and altered glucose metabolism, partly due to impaired PI3K/Akt signaling in insulin-sensitive tissues such as the liver, muscle, and adipose tissue [29, 30]. Specifically, HFD can lead to decreased PI3K activity and reduced Akt phosphorylation, which contributes to diminished glucose uptake and increased gluconeogenesis [31]. This is consistent with our previous study [32].

In this context, GPR30 may play a beneficial role by modulating the PI3K/Akt pathway to alleviate the metabolic disturbances induced by an HFD [33]. Activation of GPR30 has been shown to enhance PI3K activity and Akt phosphorylation, thereby improving insulin signaling and glucose uptake. For instance, the GPR30 agonist G-1 can improve glucose metabolism through activation of the PI3K/Akt pathway in insulin-resistant models [34].

In our study, G1 treatment may counteract the adverse effects of an HFD by activating GPR30 and subsequently stimulating the PI3K/Akt signaling pathway. This activation could restore PI3K activity that is suppressed by an HFD, leading to improved insulin sensitivity and metabolic function. By enhancing PI3K/Akt signaling, GPR30 activation might promote glucose uptake in peripheral tissues and inhibit excessive gluconeogenesis, thereby mitigating hyperglycemia and insulin resistance associated with an HFD.

However, our findings also reveal a complex role of G1 treatment. While G1 promotes lipolysis by upregulating ATGL and promoting perilipin phosphorylation, it also leads to increased levels of acetyl-CoA and enhanced activity of gluconeogenic enzymes like PEPCK and PC. This paradoxical effect suggests that GPR30 activation via G1 influences multiple metabolic pathways, some of which may exacerbate glucose metabolism disorders despite improvements in lipid metabolism.

Therefore, while GPR30 activation through the PI3K/Akt pathway may alleviate some of the negative impacts of an HFD on insulin signaling and glucose uptake, it also highlights the complexity of metabolic regulation. The interplay between enhanced lipolysis and altered glucose metabolism underscores the need for a nuanced understanding of GPR30's role in metabolic processes. Further research is necessary to elucidate the precise mechanisms by which GPR30 and the PI3K/Akt pathway interact to modulate the effects of an HFD and to determine how these pathways can be targeted for therapeutic interventions in metabolic disorders.

In conclusion, our study provides compelling evidence that the metabolic effects of G-1 are highly dependent on dietary conditions. While G-1 shows promise in reducing adiposity, its potential adverse effects on glucose metabolism under an HFD highlight the need for caution and further investigation. These findings emphasize the importance of understanding the intricate relationship between diet, metabolic pathways, and pharmacological interventions in developing effective and safe therapeutic strategies. Future research should focus on delineating the specific mechanisms by which G-1 interacts with dietary factors to modulate metabolism and exploring the potential of diet-specific treatment regimens to optimize therapeutic outcomes.

## Data Availability

The data that support the findings of this study are available from the corresponding author upon reasonable request.

## Nomenclature

ATGL: adipose triglyceride lipase
CS: citrate synthase
DEGs: differentially expressed genes
E2: estradiol
FAA: fatty acid
GLUT2: glucose transporter 2
GLUT4: glucose transporter 4
GO: Gene Ontology
GPER: the G protein–coupled estrogen receptor
GPR30: the G protein–coupled receptor 30
HFD: high-fat diet
IR: insulin resistance
KEGG: Kyoto Encyclopedia of Genes and Genomes
MRG: metabolic pathway genes
MS: metabolic syndrome
ND: normal diet
OAA: oxaloacetic acid
OGTT: oral glucose tolerance test
OMGs: obesity-associated metabolic genes
OVX: ovariectomy
PC: pyruvate carboxylase
PDK4: pyruvate dehydrogenase kinase 4
PEP: phosphoenolpyruvate
PEPCK: phosphoenolpyruvate carboxykinase
TC: total cholesterol
TG: triglyceride

## Supporting Information

Supporting Information Additional supporting information can be found online in the Supporting Information section. Figure S1. The Top 10 HUB genes related to metabolism screened from adipose tissue samples of obese menopausal women in GSE151839. (A) PCA map in GSE151839 data. (B) differential gene in heat map. (C) differential gene in volcano map. (D) metabolism-related differential genes in the Venn diagram. (E) Top 20 hub genes in PPI networks. Color depth indicates the degree of key genes from low to high. (F) Top 10 hub genes showing in the plot a boxplot. (G) Correlations analysis in top 10 hub genes. (H) KEGG analysis. Figure S2. WGCNA analysis was used to screen out the gene modules with the highest correlation with obesity, and key genes in HUB were found. (A) Each sample was grouped by obesity and normal body weight (Red/White: 0/1). (B) Hierarchical cluster analysis was performed to detect coexpression clusters with corresponding color assignments. Each color represents a module in the gene coexpression network constructed by WGCNA. (C) Modular-feature association. Each row corresponds to a module, and each column corresponds to a feature. Each cell contains the corresponding correlation and *p* value. The table is color-coded by correlation according to the color legend. (D) Sample clustering to detect outliers. All samples are located in the cluster and pass the cutoff threshold. The *x*-axis reflects the soft threshold power. The *y*-axis reflects the fitting index of the unscaled topological model. (E) The *x*-axis reflects the soft threshold power. The *y*-axis reflects average connectivity (degrees). Use soft threshold power analysis to obtain the scale-free fitting index of the network topology. (F) The heat map depicts the topological overlap matrix (TOM) of genes selected for weighted coexpression network analysis. Light colors indicate lower overlap, and red indicates higher overlap. (G) Feature gene tree and feature gene adjacency map. (H) Scatter plot describing the relationship between MM and GS in the blue module. (I) The intersection genes result from the data of GSE151839, BLUE module, metabolism, and GES26637. (J) The core genes were shown on the volcano map in the GSE26637 dataset. (K) Boxplot of core genes in the GSE26637 dataset. Figure S3. WGCNA analysis was used to screen out the gene modules with the highest correlation with insulin resistance and search for PC gene in the related modules. (A) Cluster each sample by insulin resistance and normal (Red/White: 0/1). (B) Sample clustering to detect outliers. All samples are located in the cluster and pass the cutoff threshold. *x*-axis reflects the soft threshold power. *y*-axis reflects the fitting index of the unscaled topological model. (C) The *x*-axis reflects soft threshold power. The *y*-axis reflects average connectivity (degrees). The scale-free fitting index of the network topology was obtained by soft threshold power analysis. (D) Module-feature association. Each row corresponds to a module, and each column corresponds to a feature. Each cell contains the corresponding correlation and *p* value. The table is color-coded by correlation according to the color legend. (E) Hierarchical cluster analysis was performed to detect coexpression clusters with corresponding color assignments. Each color represents a module in the gene coexpression network constructed by WGCNA. (G) The heat map depicts the topological overlap matrix (TOM) of genes selected for weighted coexpression network analysis. Light colors indicate lower overlap, and red indicates higher overlap. (H) Feature gene tree map and feature gene adjacency map. (F) Scatter plot in GREY60 module describing the relationship between MM and GS. (I) PPI network map of GREY60M module gene. Figure S4. Functional enrichment analysis. (A) GO enrichment of PC (BP). (B) GO enrichment of PC (MF). (C) GO enrichment of PC (CC). (D) KEGG enrichment of PC (GSEA) in GSE151839. (H) Verification of KEGG enrichment of PC in GSE26637 dataset. Figure S5. Insulin resistance model data supplement and G1-promoted lipolysis data supplement. (A) Display of WB results. (B) Quantification diagram of HSL protein level expression. (C) Quantification of Perilipin phosphorylation. (D) Quantification of GSK-3 expression levels in the liver. (E) Quantification of IRS1 expression in the liver.
