# Supplementary material for: GPR30 Selective Agonist G1 Exhibits Antiobesity Effects and Promotes Insulin Resistance and Gluconeogenesis in Postmenopausal Mice Fed a High-Fat Diet
Source: J Lipids. 2024 Nov 8;2024:5513473. doi: 10.1155/2024/5513473 (PMC11567725; doi:10.1155/2024/5513473)
Supplement: Supporting Information — Additional supporting information can be found online in the Supporting Information section. Figure S1. The Top 10 HUB genes related to metabolism screened from adipose tissue samples of obese menopausal women in GSE151839. (A) PCA map in GSE151839 data. (B) differential gene in heat map. (C) differential gene in volcano map. (D) metabolism-related differential genes in the Venn diagram. (E) Top 20 hub genes in PPI networks. Color depth indicates the degree of key genes from low to high. (F) Top 10 hub genes showing in the plot a boxplot. (G) Correlations analysis in top 10 hub genes. (H) KEGG analysis. Figure S2. WGCNA analysis was used to screen out the gene modules with the highest correlation with obesity, and key genes in HUB were found. (A) Each sample was grouped by obesity and normal body weight (Red/White: 0/1). (B) Hierarchical cluster analysis was performed to detect coexpression clusters with corresponding color assignments. Each color represents a module in the gene coexpression network constructed by WGCNA. (C) Modular-feature association. Each row corresponds to a module, and each column corresponds to a feature. Each cell contains the corresponding correlation and p value. The table is color-coded by correlation according to the color legend. (D) Sample clustering to detect outliers. All samples are located in the cluster and pass the cutoff threshold. The x-axis reflects the soft threshold power. The y-axis reflects the fitting index of the unscaled topological model. (E) The x-axis reflects the soft threshold power. The y-axis reflects average connectivity (degrees). Use soft threshold power analysis to obtain the scale-free fitting index of the network topology. (F) The heat map depicts the topological overlap matrix (TOM) of genes selected for weighted coexpression network analysis. Light colors indicate lower overlap, and red indicates higher overlap. (G) Feature gene tree and feature gene adjacency map. (H) Scatter plot describing the relations [file 5513473.f1.zip › supplementV3.docx]

**MATERIALS AND METHODS**

The GPR30 agonist G-1 was purchased from Cayman Chemical (Ann Arbor, MI, USA).Insulin Detection kit (batch no. CBSE05070R, CUSABIO biotechnology, Wuhan, China), Hifair® III 1st Strand cDNA Synthesis Kit(11139ES60), MolPure® Cell/Tissue Total RNA Kit(19221ES50) and Hieff® qPCR SYBR Green Master Mix(11203ES50) were bought from Yeasen Biotechnology (Shanghai, China), Phenylmethylsulfonyl fluoride (PMSF) and 5 × SDS-PAGE Sample Loading Buffer were purchased from Yeasen Biotechnology (Shanghai, China), Antibodies used in this study include the following: anti-β-actin (1:1000,YM3028,Immunoway), Phospho-HSL antibody (1:1000,YP0636,Immunoway), anti-HSL(1:8000,17333-1-AP, Proteintech ), anti-ATGL(1:1000,55190-1-AP, Proteintech ), anti-Pyruvate Caeboxylase(PC) (1:5000,16588-1-AP,Proteintech), anti-Ser522(1:1000,AF3834,Affinity),anti-HSL(1:2000,AF6403,Affinity),anti-Perilipin-1(1:2000,DF7602,Affinity). DyLight 680 (A23710), goat anti-mouse IgG and DyLight 800 (A23920), and goat anti-rabbit IgG were purchased from Abbkine (Abbkine Scientific Co., Ltd, Wuhan, China). An ELISA was performed on the serum or tissue samples to measure the levels of Triglyceride(TG, E-BC-K261-M, Elabscience), Total Cholesterol(TC, E-BC-K109-M, Elabscience) , Insulin(INS, E-EL-M1382c, Elabscience) , threonine-protein kinase GSK3B(JL18085-96T, Jonlnbio), Insulin Receptor Substrate1 (IRS1, SEC546Mu, Could-clone Corp), Glucagon(GC,E-EL-M0555c,Elabscience), Free Fatty Acids(NEFA/FFA,E-BC-K013-S,Elabscience), Phospho(enol)pyruvate (PEP, MAK102, SIGMA), Pyruvic acid(A081-1-1, COIBO BIO), Acetyl-CoA(KTB1260, Abbkine), Oxaloacetate (OAA,13840, AAT Bioquest), Citrate Synthase (CS, KTB1023, Abbkine)according to the manufacturers instructions. HE staining was performed using a HE Staining Kit (G1120, Solarbio). Oil Red staining was performed using the Lipid (Oil Red O) staining kit from Biovision according to manufacturer’s instructions. PC, GLUT2, PK, PDK4, PEPCK and GAPDH primers were obtained from Shenggong Bioengineering Technology Limited (Shanghai, China). Mouse PC forward primer CTGAAGTTCCAAACAGTTCGAGG ; reverse primer CGCACGAAACACTCGGATG. Mouse PEPCK forward primer CTGCATAACGGTCTGGACTTC; reverse primer CAGCAACTGCCCGTACTCC. Mouse PDK4 forward primer ACAGACATCATAATGTGGTCCCT ; reverse primer GGTCGATACTTCCAATGTGGC. Mouse GLUT2 forward primer TTCCAGTTCGGCTATGACATCG; reverse primer CTGGTGTGACTGTAAGTGGGG. Mouse PK forward primer GTGGCTCGGCTGAATTTCTCT; reverse primer CACCGCAACAGGACGGTAG.

**Animal models**

All animal experiments were approved by the FIRST HOSPITAL OF HEBEI MEDICAL UNIVERSITY Committee of Animal Care. The mice were fed with normal diet until 10 weeks old and then ovariectomized. After two weeks of normal diet, the mice were fed with NC/DHF diet for 6 weeks and given drugs for 6 weeks. Six mice from each group were taken before sacrifice. Experimental diets including control feed and 60% high-fat purified feed were purchased from Sano Biological Co., LTD. The product number are SN10060 and SN10010. The G-1 stoste was prepared in DMSO and then diluted into PBS. For each treated animal, 200μg G-1 was dissolved in 10μLDMSO and diluted 20 times in PBS to a final volume of 210μL. Intraperitoneal injection, 3 days a week for 6 weeks.

**Oral Glucose Tolerance Test**

Glucose tolerance test reading criteria: Normal glucose tolerance:3.9mmol/L≤FPG<6.1mmol/L, 2hPG<7.8mmol/L. Impaired fasting glucose (IFG): 6.1mmol/L≤FPG<7.0mmol/L, 2hPG<7.8mmol/L. Impaired glucose tolerance (IGT) :3.9mmol/L≤FPG<6.1mmol/L, 7.8mmol/L≤2hPG<11.1mmol/L. Diabetic glucose tolerance:7.0mmol/L≤FPG, 11.1mmol/L≤2hPG. Fasting glucose(FPG), Two-hour postprandial glucose(2hPG).

**Sample Collection and Processing**

The experimental mice were fasted for 12 h and anesthetized by intraperitoneal injection of 10% sodium ovalbumin. Subsequently, the experimental mice were executed by bleeding from the abdominal aorta. Liver tissues and visceral adipose tissues were taken from the experimental mice and stored in a refrigerator at - 80°C after rapid freezing in liquid nitrogen. The visceral adipose tissue and liver tissue of experimental mice were taken, placed in 4% paraformaldehyde for fixation and embedded in paraffin. The abdominal aortic blood specimens were placed at 4℃ for 2 h, centrifuged at 3 000 × g for 15 min, and then the serum specimens were frozen at - 80℃ for storage.

**RNA Isolation, cDNA Synthesis and Real-Time Quantitative PCR**

Approximately 40 mg of total RNA from liver tissue was extracted using the MolPure® Cell/Tissue Total RNA Kit. RNA concentration was quantified by absorbance at 260 nm on a NanoDrop Lite spectrophotometer (Thermo Fisher Scientific, Inc., USA), and RNA purity was assessed by the ratio of readings at 260 nm and 280 The RNA purity was assessed by the ratio of the readings at 260 nm and 280 nm. Reverse transcription to cDNA was performed using the FHifair® III 1st Strand cDNA Synthesis Kit (gDNA digester plus). reaction conditions: gDNA digestion at 42°C for 2 min, 25°C for 5 min, reverse transcription at 55°C for 15 min, enzyme inactivation at 85°C for 5 min. using Hieff® qPCR SYBR Green Master Mix was used for the quantification experiments, and the mixture was prepared into a 20 μl system, including 10 μl SYBR, 0,4 μl primers, 2 μl template DNA, and finally fixed with water to 20 μl. 30 sec, cycle number is 40. The RT-qPCR experiment was performed using the Talent qPCR PreMix on CFX Connect TM Real-Time System following strictly the company’sprotocol(Bio-Rad, California, United States). All procedures were carried out according the manufacturer’s protocol. The housekeeping gene, GAPDH, was used as internal reference gene, and a list of employed primers is presented in Table 1.Relative expressions of interesting genes were quantified by the method of 2−ΔΔct (Livak and Schmittgen, 2001).

**Western Blot Analysis**

0.1 g of liver tissue was removed on ice and homogenized in RIPA lysis buffer containing 1 mmol/L phenylmethylsulfonyl fluoride for 30 min, and then centrifuged at 12000 × g for 15 min at 4°C to extract total protein from the livers of experimental mice. The total protein concentration was measured on a NanoDrop Lite spectrophotometer. Total protein (50 μg) was boiled for 10 min at 100°C with the corresponding 5 × SDS-PAGE sample loading buffer and then separated by 6%-12% polyacrylamide gel electrophoresis (SDSPAGE) on a Mini-Protean Tetra cell (Bio-Rad, USA). Subsequently, the separated proteins were transferred to polyvinylidene difluoride (PVDF) membranes using Mini Trans-Blot electrophoretic transfer cells (Bio-Rad, USA) and closed with 5% skim milk (formulation: 1 mL Tween20 dissolved in 2 L TBS) prepared by TBST for 2 h. Subsequently, the PVDF membranes containing proteins were mixed with β-actin (dilution, 1 ∶:1,000), PC (dilution, 1:5,000), HSL (dilution, 1:1,000), ATGL(dilution,1:1000)and P-HSL (dilution, 1:8,000) primary antibodies were incubated overnight at 4°C. After three washes in PBST, the PVDF membranes were incubated with the corresponding fluorescent secondary antibodies (dilution 1:10 00) for 1 h at room temperature and then washed three times with TBST. The membranes were visualized using an Odyssey Imaging System (Odyssey V3.0). Quantification was performed using ImageJ v1.8.0 software (National Institutes of Health, USA).

**ELISA**

To detect changes in metabolism-related products in mouse serum and liver tissue, tissue and serum samples from each group were prepared and ELISA kits were purchased for the assays. This included the detection of free fatty acids and triglycerides in liver tissue, adipose tissue and serum. Pyruvate and phosphoenolpyruvate were assayed in liver tissue, as well as insulin, glucagon and cholesterol in serum samples. In addition, acetyl coenzyme a, oxaloacetate and citrate synthase were assayed in mitochondria extracted from liver tissues.

**Morphometric analyses**

Evaluation of lipid deposition by oil red O staining. We fixed the removed groups of liver tissues with paraformaldehyde, freeze-embedded them, and then prepared frozen sections, which were stained with the prepared Oil Red O staining solution for 2-5 min, then toned with 60% isopropyl alcohol, washed with water and re-stained with hematoxylin, divided the sections with hydrochloric acid alcohol, and finally sealed with glycerol gelatin. The Oil Red O staining was quantified using an image analysis system (Leica Q500, Cambridge, UK) and the percentage of Oil Red O staining calculated as area of Oil Red O staining relative to total analyzed area.

HE staining of the visceral adipose tissue. Fresh visceral adipose tissue from each group of mice was taken and fixed by immersion in 4% paraformaldehyde. The tissue blocks were dehydrated and then embedded with paraffin. The embedded paraffin tissues were fixed in a paraffin slicer and cut into sections with a thickness of 5-8 μm. Then HE staining was performed by placing the sections in hematoxylin solution for about 3-5 minutes, followed by color separation, water washing, then dehydration in alcohol for 10 minutes, and staining with alcohol eosin staining solution for 2-3 minutes. Finally, the sections were sealed with resin after dehydration. Adipocyte diameter was measured using ImageJ.

**Liver tissue mitochondrial extraction**

Mitochondrial isolation and protein extraction kit was purchased from Proteintech(PK10016). All operations are performed according to the instructions. The samples were stored in the -80℃ refrigerator.

**Homeostasis Model Assessment of Insulin Resistance**

Fasting blood glucose level (FPG, mmol/L) × fasting insulin level (FINS, μU/mL)/22.5. The HOMA-IR index in normal individuals is 1. As the level of insulin resistance rises, the HOMA-IR index will be higher than 1.

**Screening of Core genes**

To ensure that the network is scale-free, the soft threshold power value is chosen to be 6, and the similarity matrix is converted to the adjacency matrix. Topological overlap matrix (TOM) was constructed to measure the average network connectivity of each gene. Based on the correlation parameters (deepSplit = 2, minModuleSize = 15), the genes with similar expression profiles were divided into different modules using the dynamic tree cutting method, and the cut Height value was set to 0.85. Hierarchical clustering was used to construct a dendrogram, and the correlation between module eigengenes (MEs) and traits was calculated to screen MEs. The module with the highest correlation with obesity among all modules was identified as the most critical module for further analysis, and the intersection with the HUB genes was taken to screen out the core genes.

**Supplementary description and intext citation for figure s2,3,4,5**

**FigureS2:**

In this study, Weighted Gene Co-expression Network Analysis (WGCNA) was utilized to identify gene modules most significantly associated with obesity. Initially, samples were categorized into obese and normal weight groups (A), facilitating a clear comparative framework. Hierarchical clustering analysis was then performed, resulting in the detection of co-expression clusters represented by distinct colors, each signifying a different module within the gene co-expression network (B).[1]

The module-feature association analysis revealed varying degrees of correlation between modules and obesity-related traits. Each module's correlation coefficient and corresponding p-value were displayed, with the blue module showing the highest correlation with obesity (C). To ensure the reliability of the network, sample clustering was conducted to detect any outliers; all samples fell within the acceptable range, confirming the robustness of the data (D).[2]

Soft threshold power analysis was applied to achieve a scale-free network topology, which is crucial for accurate network construction in WGCNA. The analysis determined the optimal soft threshold power by examining the fitting index and average connectivity (E). The topological overlap matrix (TOM) heat map provided a visual representation of the network's connectivity, where lighter colors indicated lower overlap and red indicated higher overlap among genes (F).[3]

Further characterization of the modules was achieved through the feature gene tree and adjacency map, offering insights into the hierarchical relationships between genes (G). A strong positive relationship between module membership (MM) and gene significance (GS) was observed in the blue module, suggesting that genes within this module are highly relevant to obesity (H).

To pinpoint key genes, an intersection analysis was performed using data from GSE151839, the blue module, metabolic pathways, and GSE26637, resulting in a set of common genes potentially critical in obesity (I). These core genes were visualized on a volcano plot to highlight their expression differences and statistical significance (J). Boxplots further illustrated the expression levels of these core genes between obese and normal weight samples, confirming their potential role in obesity (K).

Ultimately, this comprehensive analysis led to the identification of key HUB genes within the blue module that are highly correlated with obesity, providing valuable targets for further research into the molecular mechanisms underlying obesity.

**FigureS3:**

In this study, Weighted Gene Co-expression Network Analysis (WGCNA) was employed to identify gene modules most strongly correlated with insulin resistance. The primary goal was to pinpoint modules associated with insulin resistance and to search for the PC gene within these relevant modules.Initially, samples were categorized based on insulin resistance status—insulin-resistant and normal—using a binary coding system where red represents insulin-resistant (0) and white represents normal (1) (A). This classification provided a clear framework for subsequent analysis.

To ensure data reliability, sample clustering was performed to detect any outliers. All samples were found to be within acceptable limits, passing the cutoff threshold (B). The X-axis in this analysis reflects the soft threshold power, while the Y-axis represents the fitting index of the unscaled topological model. This step is crucial for validating the quality of the data before network construction.

Soft threshold power analysis was then conducted to achieve a scale-free network topology, which is essential for the accuracy of WGCNA. The analysis involved plotting the soft threshold power against the average connectivity (degrees) (C). The optimal soft threshold power was determined by examining where the scale-free topology fitting index plateaus, indicating a balance between network complexity and scale-free topology.Module-feature association analysis was carried out to explore the relationships between different gene modules and insulin resistance (D). Each row in the resulting table corresponds to a module, and each column corresponds to a clinical feature. The cells display the correlation coefficients and corresponding p-values, color-coded according to the strength and direction of the correlation as per the legend. This analysis highlighted the modules most significantly associated with insulin resistance.Hierarchical clustering analysis was utilized to detect co-expression clusters, resulting in the identification of distinct modules represented by different colors (E). Each color signifies a unique module within the gene co-expression network constructed by WGCNA, illustrating the complex interactions among genes.The topological overlap matrix (TOM) heat map provided a visual representation of the network's connectivity (G). In this heat map, light colors indicate lower topological overlap between gene pairs, while red indicates higher overlap. This visualization helps in understanding the interconnectedness of genes within modules.

Further characterization of the modules was achieved through the feature gene tree map and adjacency map (H), which illustrate the hierarchical relationships and the strength of connections between genes within the modules.A scatter plot was generated for the GREY60 module to describe the relationship between module membership (MM) and gene significance (GS) (F). A strong positive correlation in this plot indicates that genes highly connected within the module are also highly significant with respect to insulin resistance. This suggests that the GREY60 module is particularly relevant to the condition.

Finally, a protein-protein interaction (PPI) network map was constructed for the genes within the GREY60 module (I). This network map helps in visualizing the interactions at the protein level, providing insights into the biological processes and pathways involved in insulin resistance.

Through this comprehensive analysis, the GREY60 module was identified as having the highest correlation with insulin resistance. The PC gene was found within this module, suggesting its potential role in the molecular mechanisms underlying insulin resistance. These findings offer valuable targets for further research and potential therapeutic interventions for insulin resistance.

**FigureS4:**

In this study, functional enrichment analysis was performed to elucidate the biological roles and significance of the PC gene within the datasets analyzed. The analysis comprised both Gene Ontology (GO) enrichment and Kyoto Encyclopedia of Genes and Genomes (KEGG) pathway enrichment, offering a comprehensive view of the gene's involvement in various biological contexts.[4]

Firstly, GO enrichment analysis was conducted for the PC gene across three main categories: Biological Process (BP), Molecular Function (MF), and Cellular Component (CC). In the Biological Process category (A), the analysis identified key processes that the PC gene is associated with, highlighting its role in essential biological activities. The Molecular Function enrichment (B) revealed the specific biochemical activities and interactions that PC is involved in at the molecular level. The Cellular Component analysis (C) provided insights into the specific cellular locations where PC is active, indicating where within the cell the gene exerts its functions.

Next, KEGG pathway enrichment analysis was performed using Gene Set Enrichment Analysis (GSEA) on the GSE151839 dataset (D). This analysis aimed to uncover significant pathways that are enriched with the PC gene, shedding light on the broader biological pathways and networks in which PC participates. The GSEA approach allowed for the identification of pathways that are significantly associated with the expression levels of PC, offering a pathway-centric perspective on its potential roles in biological processes and disease mechanisms.

To validate the KEGG enrichment findings from the GSE151839 dataset, a verification step was carried out using the GSE26637 dataset (H). [5,6] This verification is crucial for confirming the reproducibility and consistency of the enrichment results across independent datasets. By demonstrating that the KEGG pathways enriched for PC in GSE151839 are also significant in GSE26637, the study strengthens the evidence for the PC gene's involvement in these pathways.

Overall, the functional enrichment analysis provided a detailed understanding of the PC gene's roles at the biological process, molecular function, and cellular component levels, as well as its participation in key biological pathways. These findings contribute valuable insights into the molecular mechanisms underlying the conditions studied and highlight the potential of PC as a significant gene of interest for further research and therapeutic exploration.

References:

[1] Langfelder P, Horvath S. WGCNA: an R package for weighted correlation network analysis. BMC Bioinformatics. 2008 Dec 29;9:559. doi: 10.1186/1471-2105-9-559.

[2] Zhang B, Horvath S. A general framework for weighted gene co-expression network analysis. STAT APPL GENET MOL 2005;4:Article17.

[3] Szklarczyk D, Gable AL, Lyon D, Junge A, Wyder S, Huerta-Cepas J, Simonovic M, Doncheva NT, Morris JH, Bork P, Jensen LJ, Mering CV. STRING v11: protein-protein association networks with increased coverage, supporting functional discovery in genome-wide experimental datasets. NUCLEIC ACIDS RES 2019;47:D607-13.

[4] Huang DW, Lempicki RA, Sherman BT. Systematic and integrative analysis of large gene lists using DAVID bioinformatics resources. NAT PROTOC 2009;4:44-57.

[5] Subramanian A, Tamayo P, Mootha VK, Mukherjee S, Ebert BL, Gillette MA, Paulovich A, Pomeroy SL, Golub TR, Lander ES, Mesirov JP. Gene set enrichment analysis: a knowledge-based approach for interpreting genome-wide expression profiles. P NATL ACAD SCI USA 2005;102:15545-50.

[6] Kanehisa M, Sato Y, Kawashima M, Furumichi M, Tanabe M. KEGG as a reference resource for gene and protein annotation. NUCLEIC ACIDS RES 2016;44:D457-62.
