# Supplementary figures and images for: GPR30 Selective Agonist G1 Exhibits Antiobesity Effects and Promotes Insulin Resistance and Gluconeogenesis in Postmenopausal Mice Fed a High-Fat Diet
Source: J Lipids. 2024 Nov 8;2024:5513473. doi: 10.1155/2024/5513473 (PMC11567725; doi:10.1155/2024/5513473)

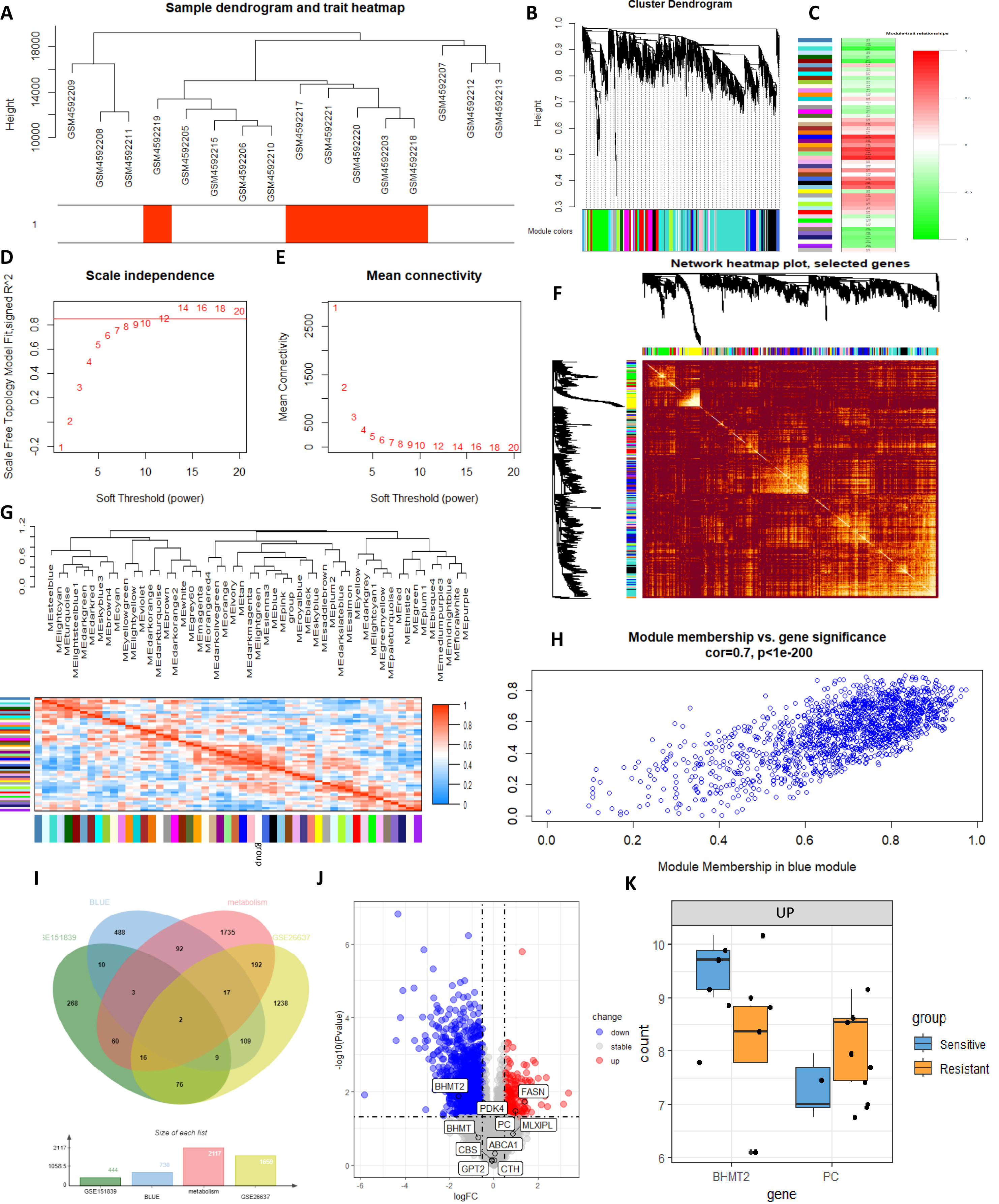

Liu et al. FigureS2

Supplement: Supporting Information — Additional supporting information can be found online in the Supporting Information section. Figure S1. The Top 10 HUB genes related to metabolism screened from adipose tissue samples of obese menopausal women in GSE151839. (A) PCA map in GSE151839 data. (B) differential gene in heat map. (C) differential gene in volcano map. (D) metabolism-related differential genes in the Venn diagram. (E) Top 20 hub genes in PPI networks. Color depth indicates the degree of key genes from low to high. (F) Top 10 hub genes showing in the plot a boxplot. (G) Correlations analysis in top 10 hub genes. (H) KEGG analysis. Figure S2. WGCNA analysis was used to screen out the gene modules with the highest correlation with obesity, and key genes in HUB were found. (A) Each sample was grouped by obesity and normal body weight (Red/White: 0/1). (B) Hierarchical cluster analysis was performed to detect coexpression clusters with corresponding color assignments. Each color represents a module in the gene coexpression network constructed by WGCNA. (C) Modular-feature association. Each row corresponds to a module, and each column corresponds to a feature. Each cell contains the corresponding correlation and p value. The table is color-coded by correlation according to the color legend. (D) Sample clustering to detect outliers. All samples are located in the cluster and pass the cutoff threshold. The x-axis reflects the soft threshold power. The y-axis reflects the fitting index of the unscaled topological model. (E) The x-axis reflects the soft threshold power. The y-axis reflects average connectivity (degrees). Use soft threshold power analysis to obtain the scale-free fitting index of the network topology. (F) The heat map depicts the topological overlap matrix (TOM) of genes selected for weighted coexpression network analysis. Light colors indicate lower overlap, and red indicates higher overlap. (G) Feature gene tree and feature gene adjacency map. (H) Scatter plot describing the relations [file 5513473.f1.zip › supplement figure2.pdf]

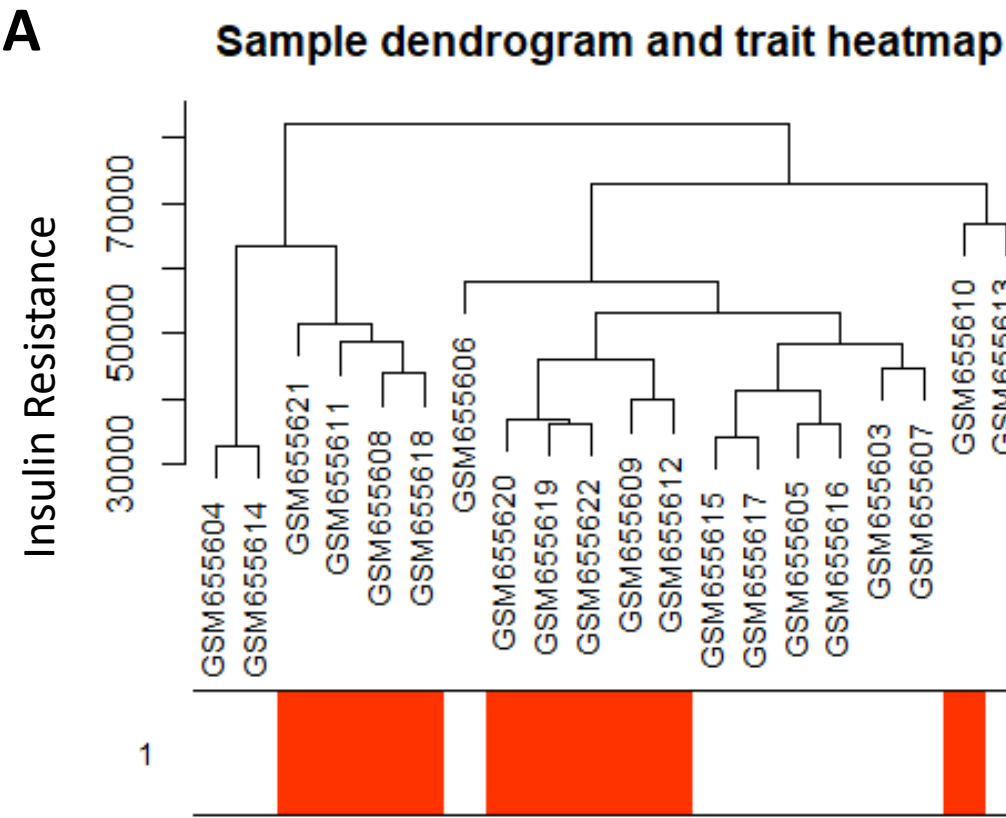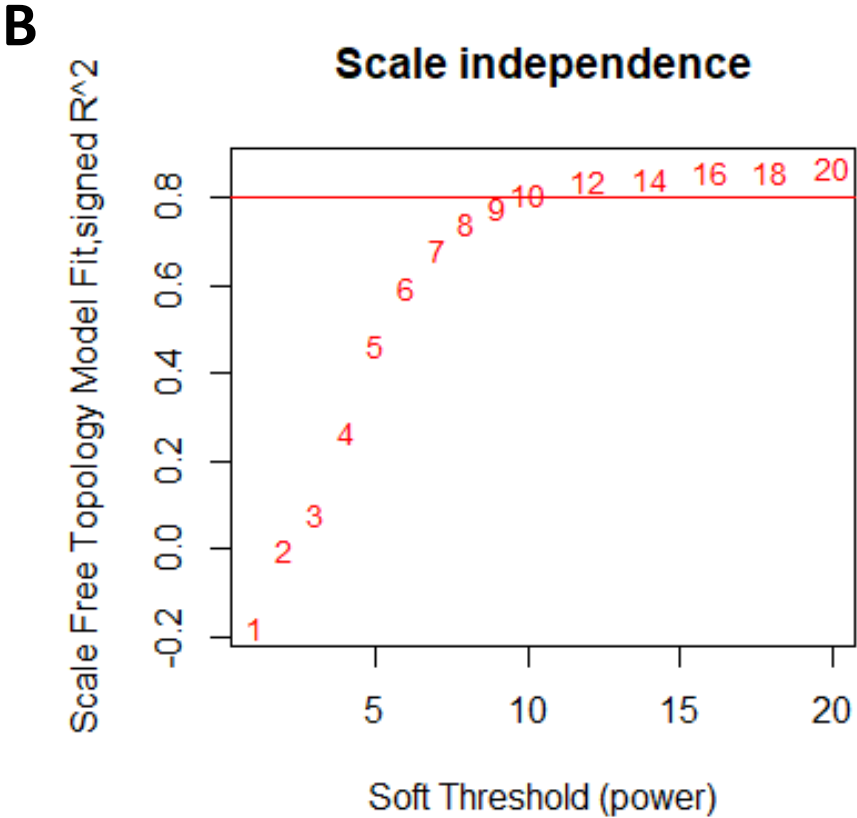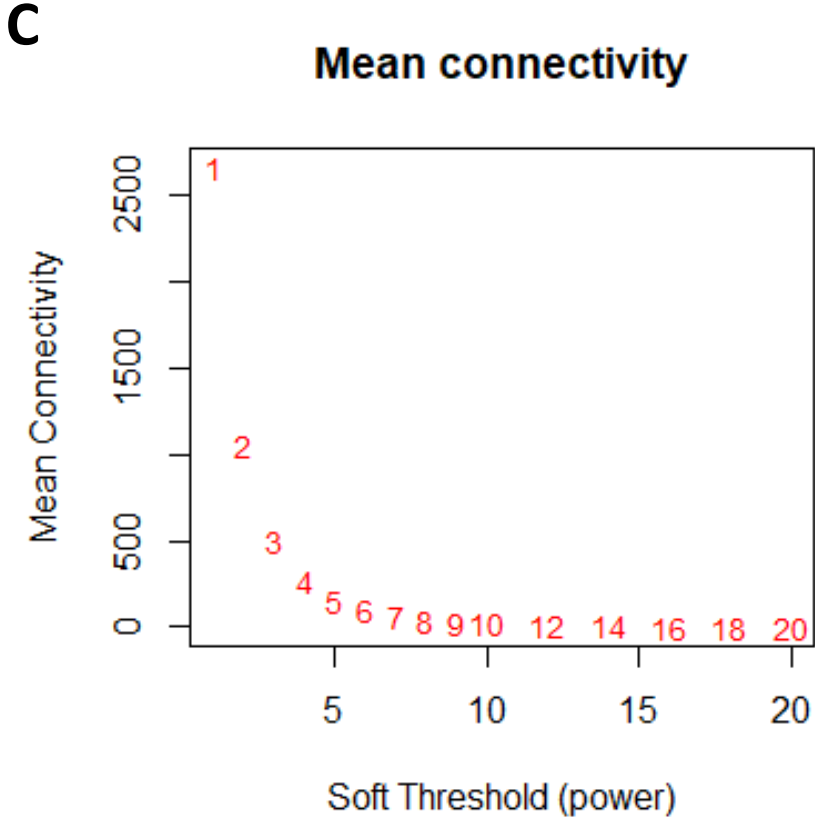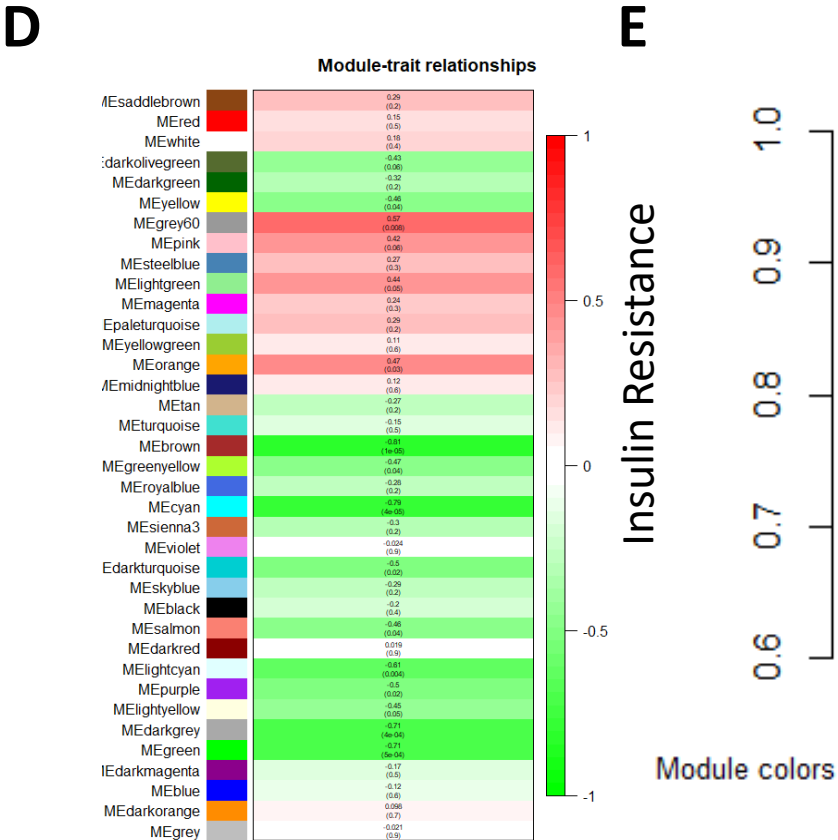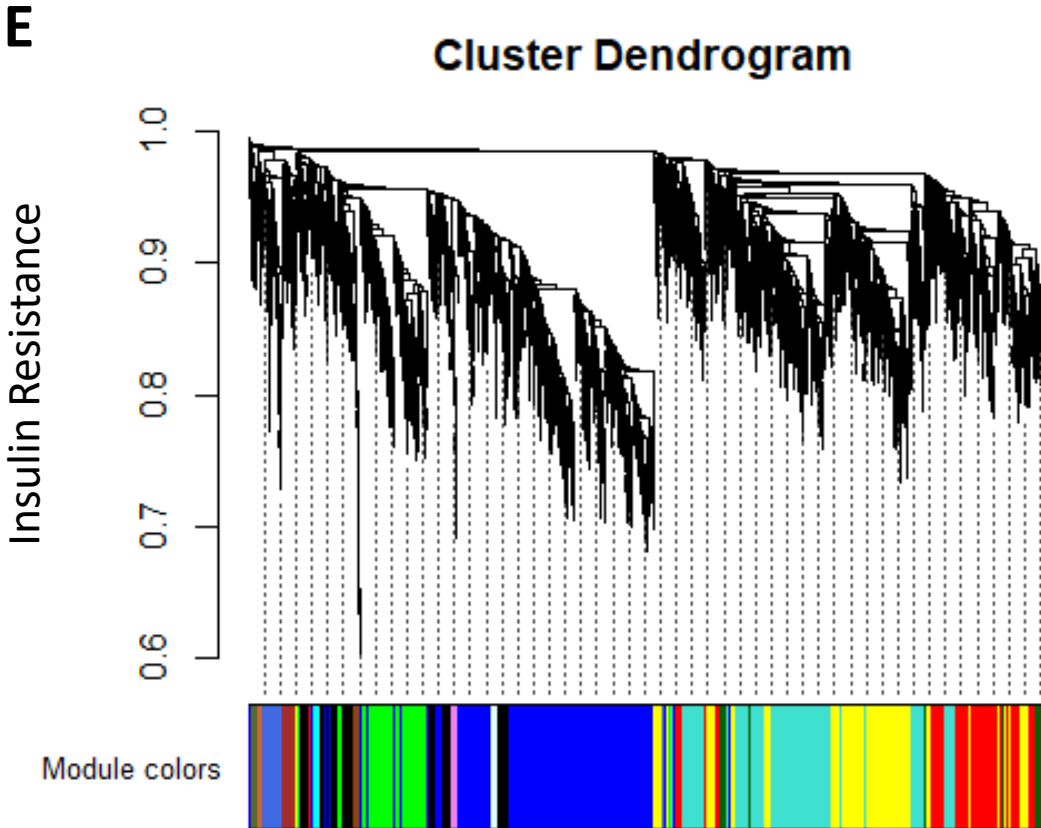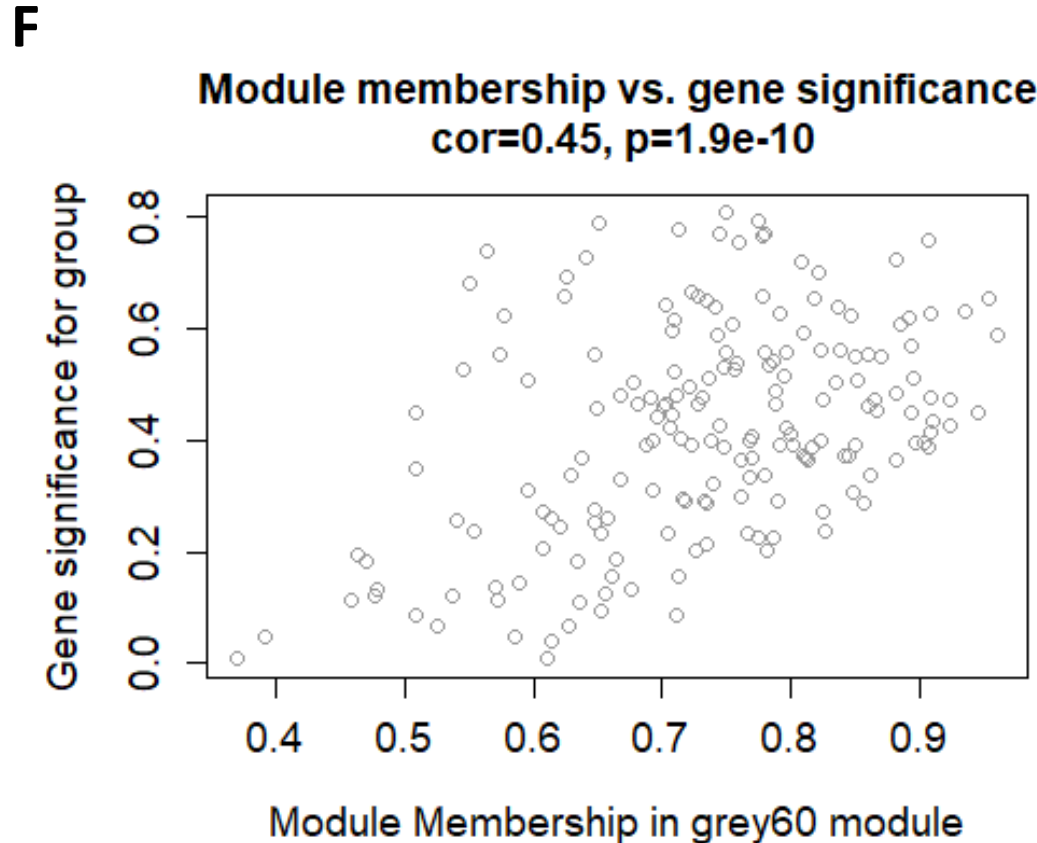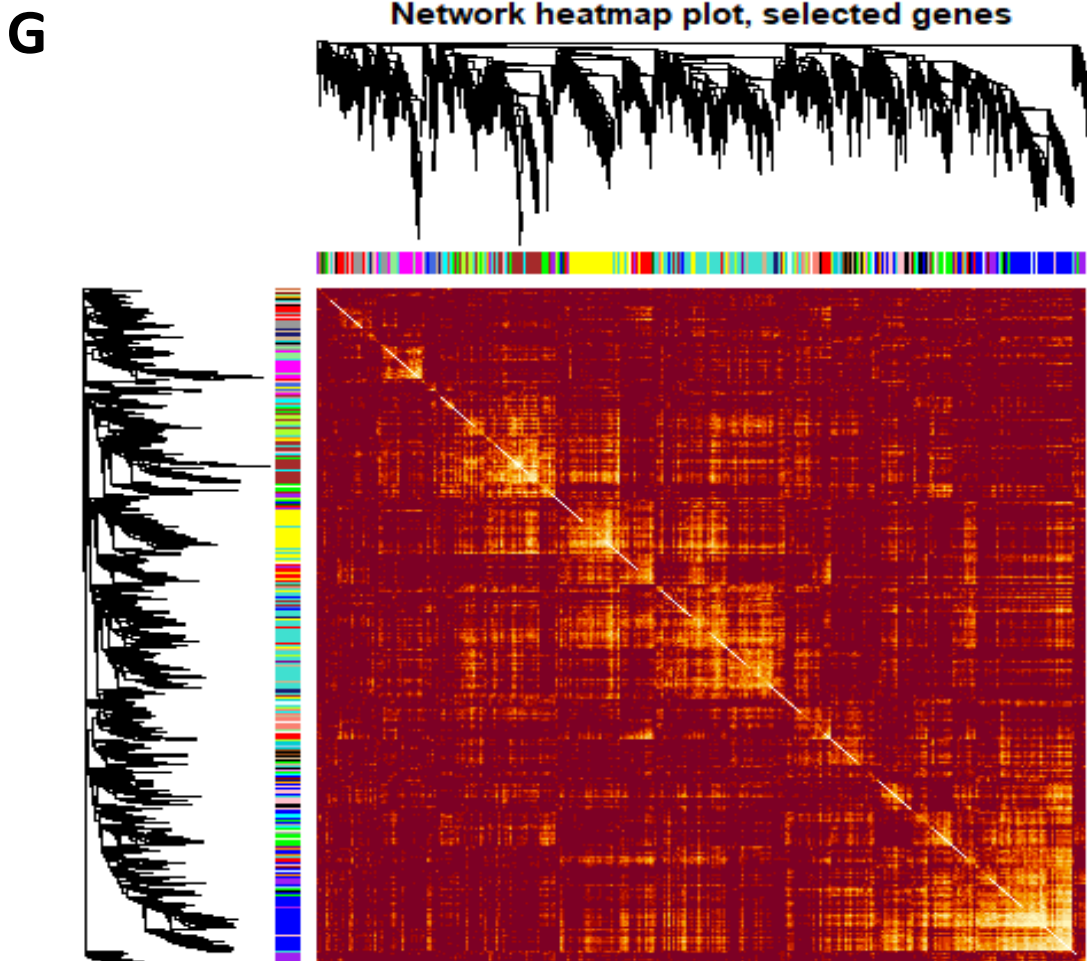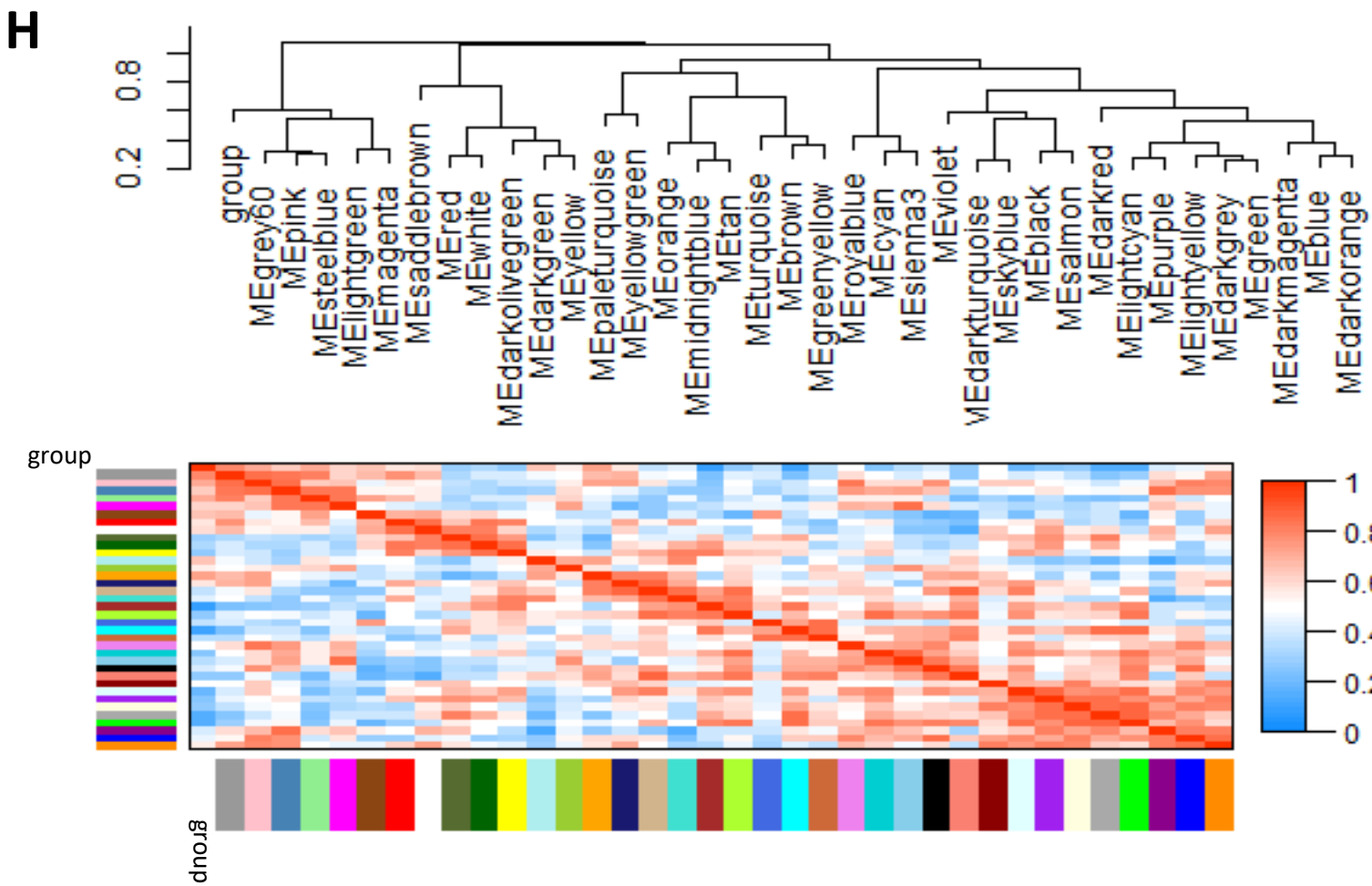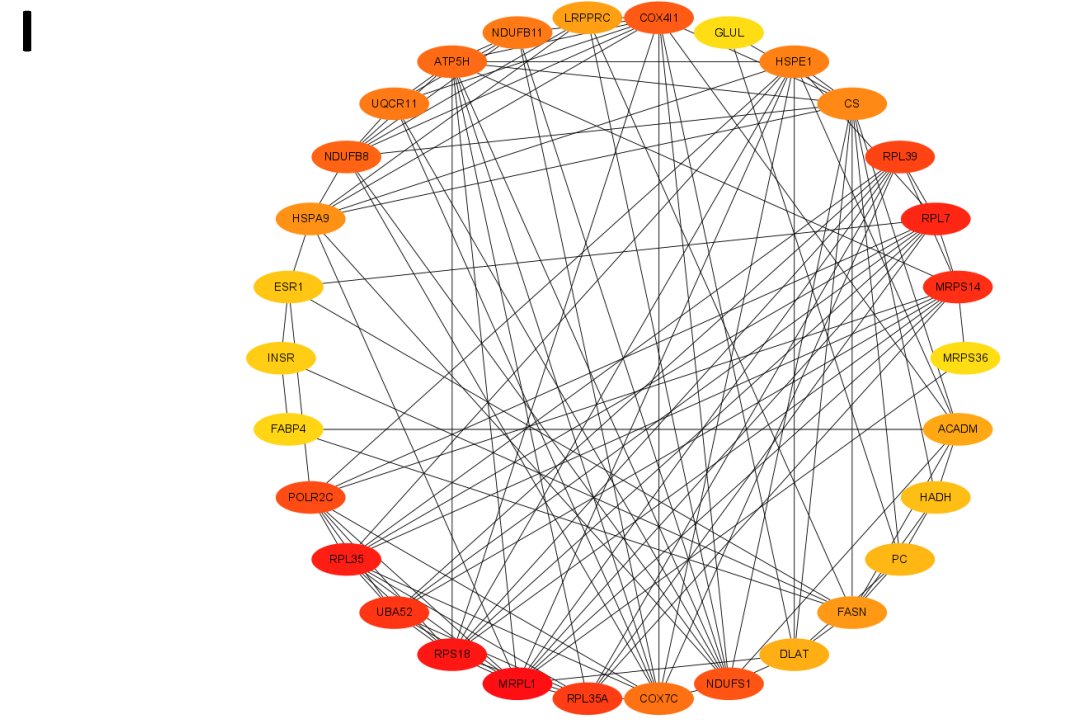

Supplement: Supporting Information — Additional supporting information can be found online in the Supporting Information section. Figure S1. The Top 10 HUB genes related to metabolism screened from adipose tissue samples of obese menopausal women in GSE151839. (A) PCA map in GSE151839 data. (B) differential gene in heat map. (C) differential gene in volcano map. (D) metabolism-related differential genes in the Venn diagram. (E) Top 20 hub genes in PPI networks. Color depth indicates the degree of key genes from low to high. (F) Top 10 hub genes showing in the plot a boxplot. (G) Correlations analysis in top 10 hub genes. (H) KEGG analysis. Figure S2. WGCNA analysis was used to screen out the gene modules with the highest correlation with obesity, and key genes in HUB were found. (A) Each sample was grouped by obesity and normal body weight (Red/White: 0/1). (B) Hierarchical cluster analysis was performed to detect coexpression clusters with corresponding color assignments. Each color represents a module in the gene coexpression network constructed by WGCNA. (C) Modular-feature association. Each row corresponds to a module, and each column corresponds to a feature. Each cell contains the corresponding correlation and p value. The table is color-coded by correlation according to the color legend. (D) Sample clustering to detect outliers. All samples are located in the cluster and pass the cutoff threshold. The x-axis reflects the soft threshold power. The y-axis reflects the fitting index of the unscaled topological model. (E) The x-axis reflects the soft threshold power. The y-axis reflects average connectivity (degrees). Use soft threshold power analysis to obtain the scale-free fitting index of the network topology. (F) The heat map depicts the topological overlap matrix (TOM) of genes selected for weighted coexpression network analysis. Light colors indicate lower overlap, and red indicates higher overlap. (G) Feature gene tree and feature gene adjacency map. (H) Scatter plot describing the relations [file 5513473.f1.zip › supplement figure3.pdf]

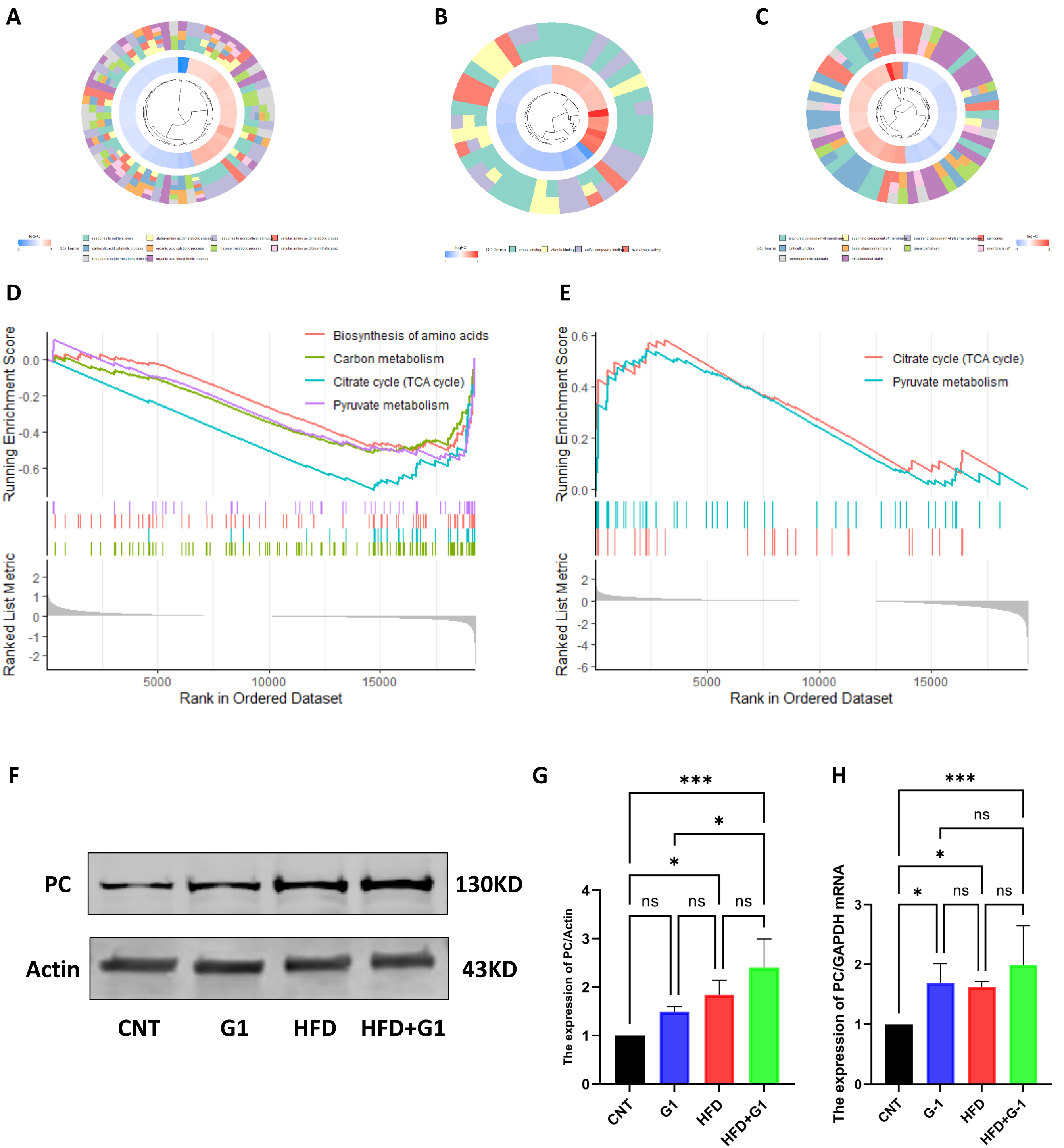

Supplement: Supporting Information — Additional supporting information can be found online in the Supporting Information section. Figure S1. The Top 10 HUB genes related to metabolism screened from adipose tissue samples of obese menopausal women in GSE151839. (A) PCA map in GSE151839 data. (B) differential gene in heat map. (C) differential gene in volcano map. (D) metabolism-related differential genes in the Venn diagram. (E) Top 20 hub genes in PPI networks. Color depth indicates the degree of key genes from low to high. (F) Top 10 hub genes showing in the plot a boxplot. (G) Correlations analysis in top 10 hub genes. (H) KEGG analysis. Figure S2. WGCNA analysis was used to screen out the gene modules with the highest correlation with obesity, and key genes in HUB were found. (A) Each sample was grouped by obesity and normal body weight (Red/White: 0/1). (B) Hierarchical cluster analysis was performed to detect coexpression clusters with corresponding color assignments. Each color represents a module in the gene coexpression network constructed by WGCNA. (C) Modular-feature association. Each row corresponds to a module, and each column corresponds to a feature. Each cell contains the corresponding correlation and p value. The table is color-coded by correlation according to the color legend. (D) Sample clustering to detect outliers. All samples are located in the cluster and pass the cutoff threshold. The x-axis reflects the soft threshold power. The y-axis reflects the fitting index of the unscaled topological model. (E) The x-axis reflects the soft threshold power. The y-axis reflects average connectivity (degrees). Use soft threshold power analysis to obtain the scale-free fitting index of the network topology. (F) The heat map depicts the topological overlap matrix (TOM) of genes selected for weighted coexpression network analysis. Light colors indicate lower overlap, and red indicates higher overlap. (G) Feature gene tree and feature gene adjacency map. (H) Scatter plot describing the relations [file 5513473.f1.zip › supplement figure4.pdf]

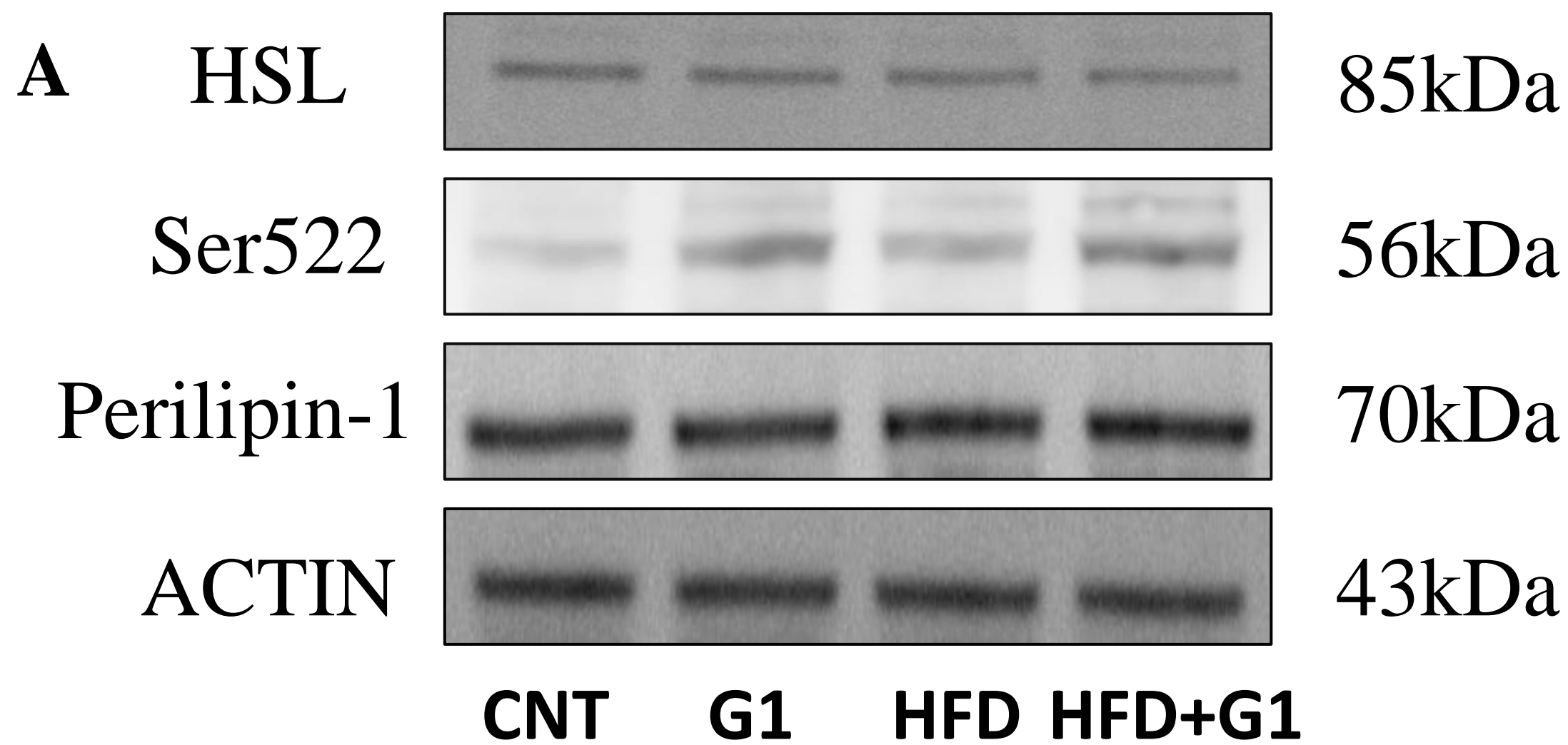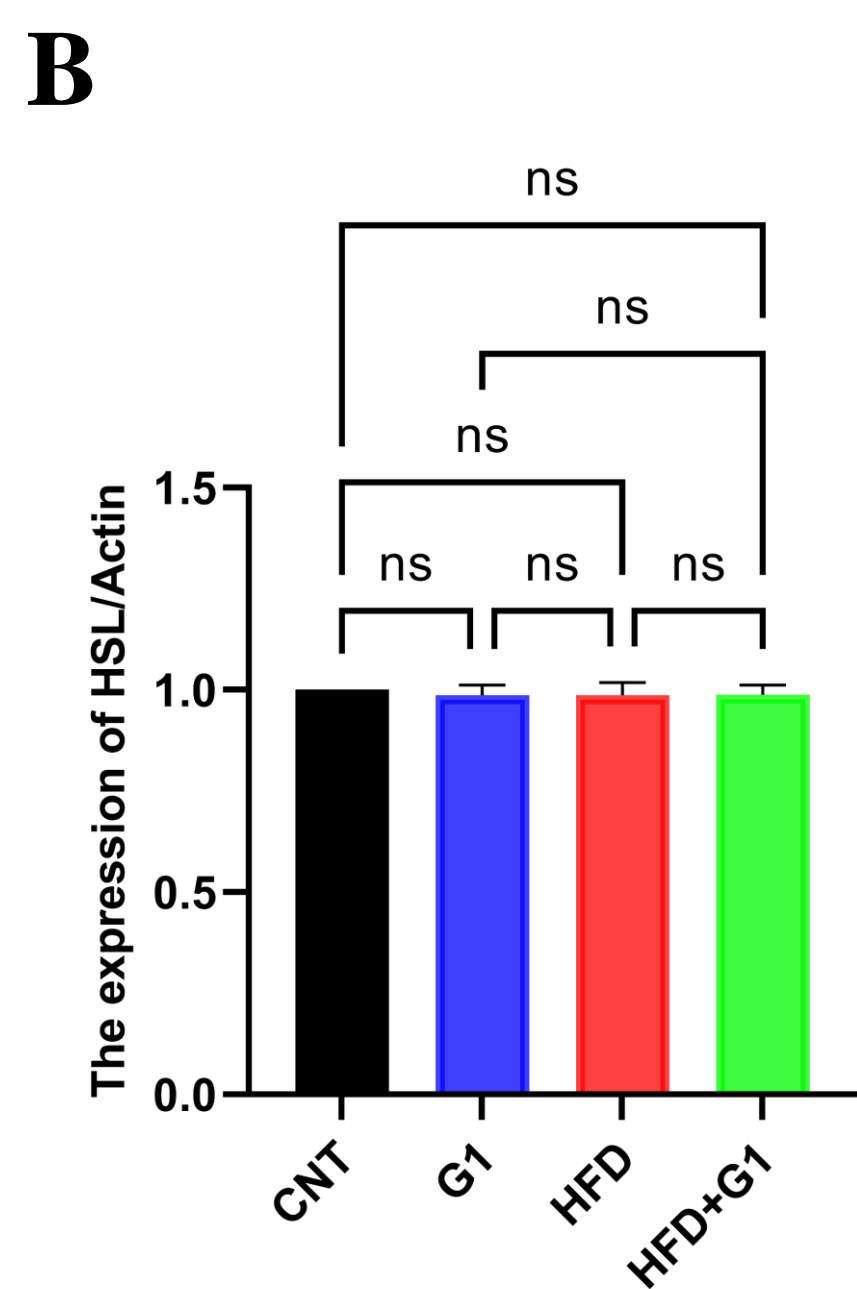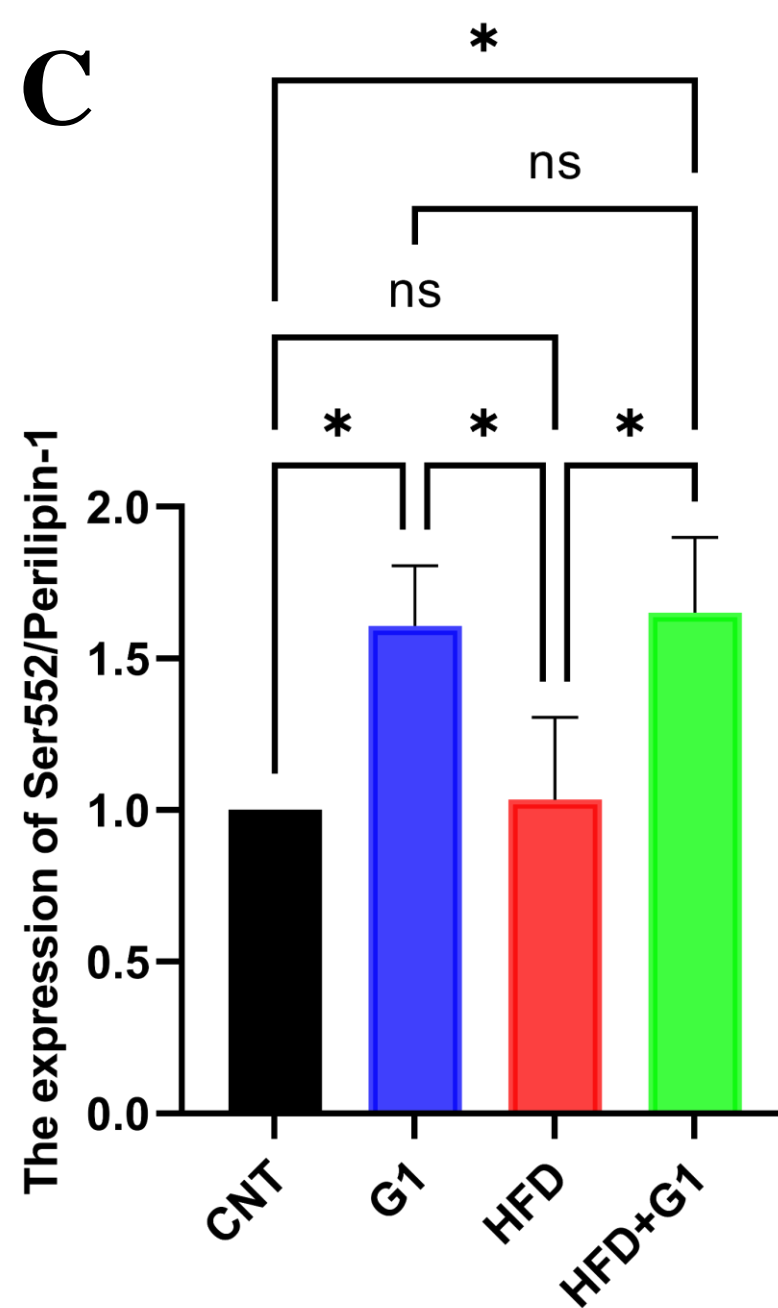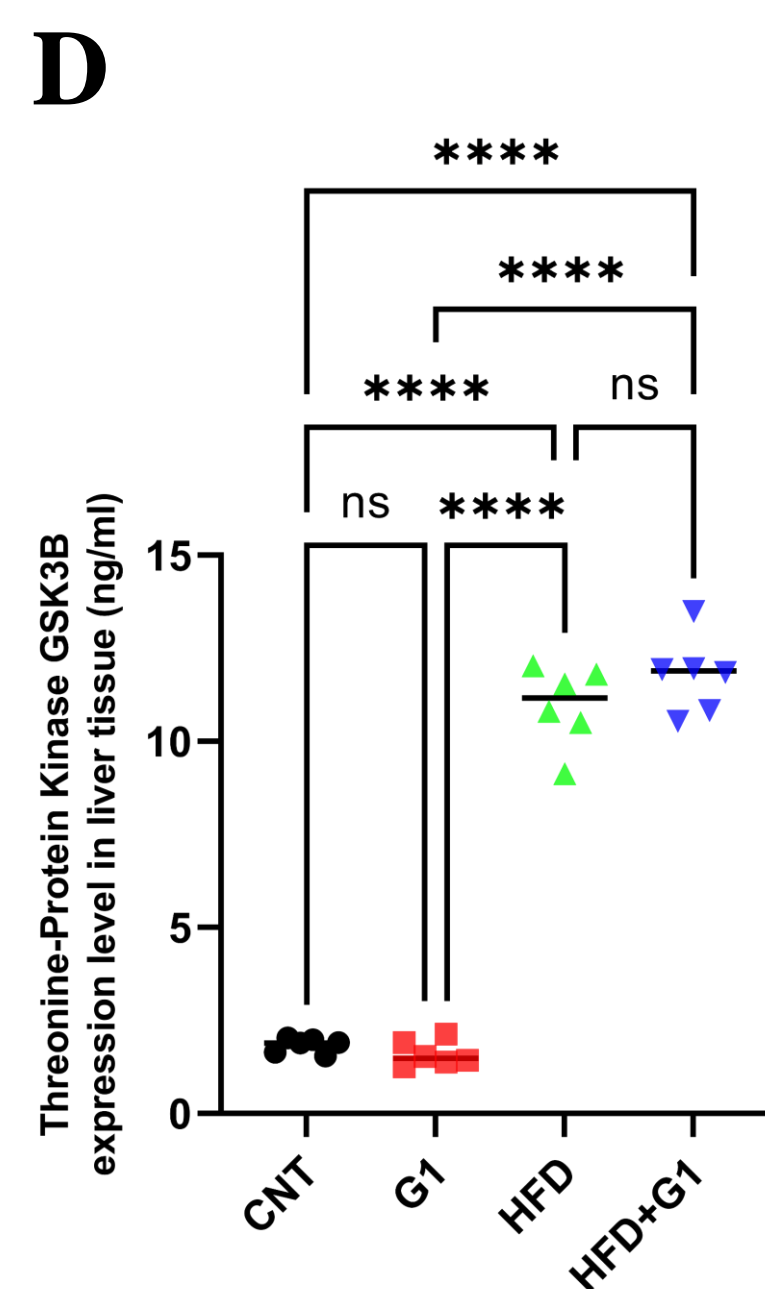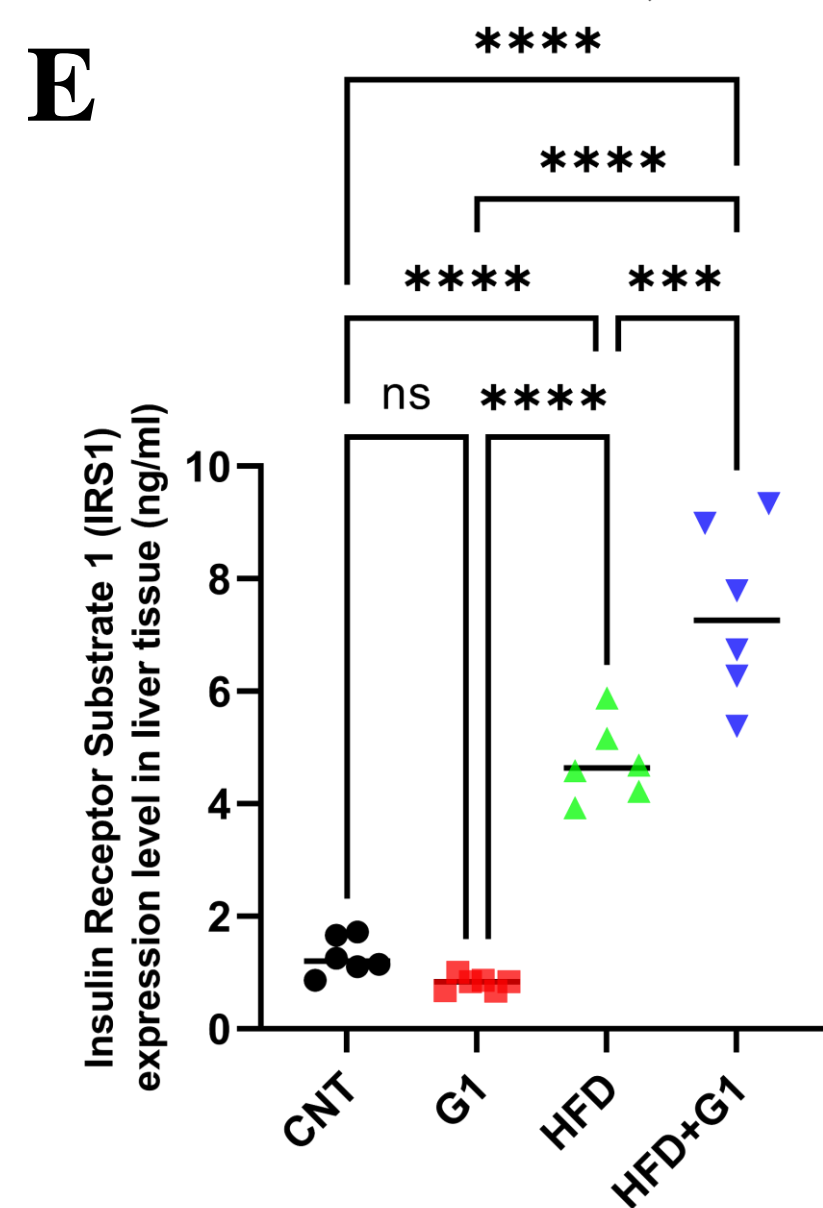

Supplement: Supporting Information — Additional supporting information can be found online in the Supporting Information section. Figure S1. The Top 10 HUB genes related to metabolism screened from adipose tissue samples of obese menopausal women in GSE151839. (A) PCA map in GSE151839 data. (B) differential gene in heat map. (C) differential gene in volcano map. (D) metabolism-related differential genes in the Venn diagram. (E) Top 20 hub genes in PPI networks. Color depth indicates the degree of key genes from low to high. (F) Top 10 hub genes showing in the plot a boxplot. (G) Correlations analysis in top 10 hub genes. (H) KEGG analysis. Figure S2. WGCNA analysis was used to screen out the gene modules with the highest correlation with obesity, and key genes in HUB were found. (A) Each sample was grouped by obesity and normal body weight (Red/White: 0/1). (B) Hierarchical cluster analysis was performed to detect coexpression clusters with corresponding color assignments. Each color represents a module in the gene coexpression network constructed by WGCNA. (C) Modular-feature association. Each row corresponds to a module, and each column corresponds to a feature. Each cell contains the corresponding correlation and p value. The table is color-coded by correlation according to the color legend. (D) Sample clustering to detect outliers. All samples are located in the cluster and pass the cutoff threshold. The x-axis reflects the soft threshold power. The y-axis reflects the fitting index of the unscaled topological model. (E) The x-axis reflects the soft threshold power. The y-axis reflects average connectivity (degrees). Use soft threshold power analysis to obtain the scale-free fitting index of the network topology. (F) The heat map depicts the topological overlap matrix (TOM) of genes selected for weighted coexpression network analysis. Light colors indicate lower overlap, and red indicates higher overlap. (G) Feature gene tree and feature gene adjacency map. (H) Scatter plot describing the relations [file 5513473.f1.zip › supplement figure5.pdf]
